# Graph-based clustering and characterization of repetitive sequences in next-generation sequencing data

**DOI:** 10.1186/1471-2105-11-378

**Published:** 2010-07-15

**Authors:** Petr Novák, Pavel Neumann, Jiří Macas

**Affiliations:** 1 Biology Centre ASCR, Institute of Plant Molecular Biology, Branisovska 31, Ceske Budejovice, CZ-37005, Czech Republic

## Abstract

**Background:**

The investigation of plant genome structure and evolution requires comprehensive characterization of repetitive sequences that make up the majority of higher plant nuclear DNA. Since genome-wide characterization of repetitive elements is complicated by their high abundance and diversity, novel approaches based on massively-parallel sequencing are being adapted to facilitate the analysis. It has recently been demonstrated that the low-pass genome sequencing provided by a single 454 sequencing reaction is sufficient to capture information about all major repeat families, thus providing the opportunity for efficient repeat investigation in a wide range of species. However, the development of appropriate data mining tools is required in order to fully utilize this sequencing data for repeat characterization.

**Results:**

We adapted a graph-based approach for similarity-based partitioning of whole genome 454 sequence reads in order to build clusters made of the reads derived from individual repeat families. The information about cluster sizes was utilized for assessing the proportion and composition of repeats in the genomes of two model species, *Pisum sativum *and *Glycine max*, differing in genome size and 454 sequencing coverage. Moreover, statistical analysis and visual inspection of the topology of the cluster graphs using a newly developed program tool, *SeqGrapheR*, were shown to be helpful in distinguishing basic types of repeats and investigating sequence variability within repeat families.

**Conclusions:**

Repetitive regions of plant genomes can be efficiently characterized by the presented graph-based analysis and the graph representation of repeats can be further used to assess the variability and evolutionary divergence of repeat families, discover and characterize novel elements, and aid in subsequent assembly of their consensus sequences.

## Background

The ability of next-generation sequencing technologies to analyze eukaryotic genomes in a fast and cost-efficient manner [1-3] is providing new opportunities for investigating biological problems that, due to their complexity, could not be addressed before. One such question concerns the role that repetitive DNA plays in shaping the structure and evolution of plant genomes. Its elucidation depends in large part on performing a comparative analysis of repeat composition in a large number of plant species differing in size and other characteristics of their genomes. However, repetitive sequences, composed of numerous and diverse families of mobile elements and tandem repeats, account for up to 97% of plant nuclear DNA [4,5]. Thus, genome-wide characterization of repetitive elements can only be achieved when large volumes of sequencing data are available, which has long been limited to a few model species due to the speed and cost constraints imposed by classical sequencing. Compared to the conventional, clone-based Sanger sequencing approaches, the next-generation technologies work at unprecedented speed, sequencing up to several gigabases in a single reaction for a fraction of the cost [1-3]. Although this amount of sequencing data is still not sufficient to provide the coverage typically needed for whole genome assembly, it enables representative sampling of elements present in a genome in multiple copies. For example, a low-pass sequencing providing only 0.008 × coverage of the pea (*Pisum sativum*) genome was found to efficiently capture repetitive sequences present in the genome with at least 1000 copies. Moreover, the proportion of individual sequences in the reads reflected their genomic abundance, thus providing a simple and reliable means for quantification of repetitive elements [6].

The potential of bioinformatic analysis of low-depth sequencing data for plant repeat investigation has been further demonstrated in several studies. For instance, the identification of BAC clone regions representing soybean genomic repeats was achieved by quantification of the number of similarity hits to a database of the soybean (*Glycine max*) whole-genome 454 reads [7]. An alternative approach was adapted for repeat detection in barley clones, using data from Solexa/Illumina sequencing. In this case, the genome sequence reads were decomposed to 20-mers and their summarized frequencies were used to build an index of Mathematically Defined Repeats, which was then employed to detect repetitive regions [8]. While these applications utilize the sequencing data only for repeat content evaluation in reference genomic sequences, there is also the possibility of performing *de novo *repeat identification and reconstruction solely from the sequence reads. This can be achieved by direct assembly of the reads, as has been reported for soybean, where 41% of 717,383 genomic 454 reads were assembled into contigs using the phrap program [7]. Due to the low genome coverage of the sequencing, most of the contigs did not represent specific genomic loci; instead, they were composed of reads derived from multiple copies of repetitive elements, thus representing prototype (or consensus) sequences of genomic repeats. Even though the exact form of this consensus does not necessarily occur in the genome, this representation of repetitive elements is sufficiently accurate to enable amplification of the whole length repetitive elements using PCR [7]. The contigs could then be used to evaluate the abundance of their corresponding genomic sequences based on the number of assembled reads, and some of them could be classified based on their similarity to known repetitive elements.

Another approach for repeat identification and quantification was introduced in a study of the pea genome by 454 sequencing and subsequent clustering analysis of the reads [6]. This analysis was based on an all-to-all comparison of sequence reads to reveal their similarities, which were used to build clusters of overlapping reads representing different repetitive elements. Information about cluster sizes (numbers of reads within the clusters) was used to quantify individual repeat families, leading to characterization of repeats representing up to 48% of the pea genome. As there is considerable sequence variability in genomic copies of repeated elements, their assembly typically includes only part of their reads and results in multiple contigs. Thus, the advantage of the cluster-based quantification over the contig-based approach is that evaluation of individual reads better captures repeat variability and is therefore more informative. Subsequent contig assembly is then also possible, and it is made computationally less-demanding and suitable for parallelization, because it is performed within individual clusters instead of the whole set of reads.

While the clustering-based repeat analysis has proved to be principally sound, its initial implementation using the tclust program [9] suffered from the formation of chimeric clusters made of several unrelated families of high-copy elements [6]. Tclust employs a simple transitive-closure clustering algorithm, which is well suited for its original purpose of clustering EST sequences [9]. However, when applied to whole genome sequencing it is prone to producing mixed clusters due to the occurrence of "bridge" reads with partial similarity to two groups of unrelated sequences. Such reads can presumably originate from insertion sites of mobile elements or from structural and regulatory sequences conserved across diverse families of elements. To overcome these limitations, we focused on developing more sophisticated approaches facilitating precise repeat clustering and analysis.

In this work, we describe the principles and implementation of graph-based methods for similarity-based clustering of sequence reads and further analysis of sequence clusters. These methods were applied to real datasets of 454 reads from soybean (*Glycine max*) and pea (*Pisum sativum*), chosen to represent different-sized genomes and different sequencing coverage. Moreover, these datasets have already been investigated [6,7], thus allowing comparison of the newly developed methods to those used previously. We demonstrate that our methods provide several advantages over previous approaches, including improved partitioning of different types of repetitive elements. In addition, the analysis of the graph structure of sequence clusters enables partial classification of sequences without prior knowledge of sequence information, which can be specifically helpful in the characterization of novel repeats from poorly characterized genomes.

## Results

### Principles of graph-based clustering of sequence reads

**Figure 1:**
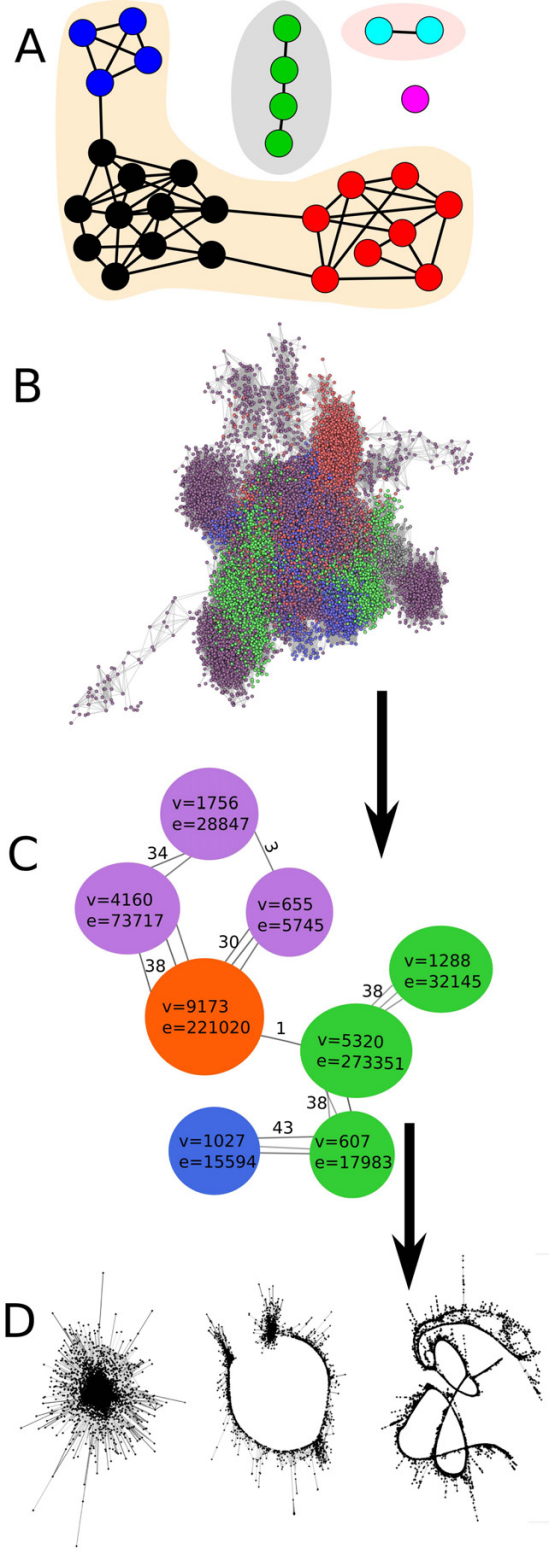
**Sequence reads organized in a graph structure**. Single reads are represented by vertices (nodes) and their sequence overlaps by edges **(A) Examples of different types of clusters that can be found in the graph structure**. Graph parts in the shaded areas represent connected components of the graph. Nodes with the same color correspond to clusters (communities) as identified using a hierarchical agglomeration algorithm. In some cases connected components are identical to clusters identified by the hierarchical agglomeration method (green nodes in gray shading and turquoise nodes in pink shading). Magenta node represents a singlet - a read with no similarity to other sequences. **(B-D) Principles of sequence read clustering and cluster analysis**. (B) An example of a graph built from reads sampled from the largest connected component of *P. sativum*. Communities of reads were identified by the hierarchical agglomeration algorithm and labeled to distinguish different classes of repeats. (C) Schematic representation of resulting clusters (colored circles), showing number of reads (v) and number of edges (e) within and between the clusters. (D) Graph layouts calculated using the Fruchterman and Reingold algorithm for three clusters differing in structure.

The analysis is performed on a set of 454 sequence reads, representing short nucleotide sequences randomly sampled from the analyzed genome. It starts with the identification of read similarities by performing all-to-all pairwise comparisons and recording all read pairs with sequence overlaps exceeding a specified threshold. This information is then used to construct a graph in which the vertices correspond to sequence reads, overlapping reads are connected with edges and their similarity score is expressed as an edge weight. The graph construction, its corresponding data structures and subsequent analysis are implemented in the R programming environment [10-12]. A simplified example of a graph is given in Fig. 1A, showing various features of the graph structure. In the case of low-depth sequencing, providing less than 0.5× genome coverage, single-copy sequences are only sparsely covered and thus overlaps of their reads are rare, resulting in isolated nodes with no connections to other parts of the graph. On the other hand, repetitive sequences constitute groups of mutually connected nodes, due to frequent sequence overlaps of reads pooled from their multiple copies. An isolated group of nodes in which any two vertices are connected by a path and no more vertices or edges can be added is termed a *connected component *(Fig. 1A). Identification of connected components is a principle of sequence clustering performed by the tclust program [9] that has previously been employed for repeat clustering analysis [6]. Ideally, such clustering should be sufficient to separate sequence reads originating from different families of repetitive elements. In reality, frequent interspersion and partial sequence similarities of genomic repeats lead to merged (connected) clusters including multiple distinct elements, especially as the repeat abundance and/or sequencing coverage increases. To deal with this problem, we performed further analysis of the graph structure using a hierarchical agglomeration algorithm [13] for detecting *communities *[14] which are defined as groups of vertices in a graph that are more densely connected internally than with the rest of the graph (Fig. 1A, B). To find the optimal graph partitioning into these communities, the greedy algorithm is used to find graph divisions into subgraphs with the maximal *modularity*. Briefly, modularity, which is the quality measure for graph clustering, is used to evaluate the frequency of node connections within the same community with respect to the value that is expected for a randomly connected graph [15,16]. If the number of edges within a communities is no better than random, then the modularity of that division into communities is zero, whereas a modularity close to 1 indicates strong community structure. Analysis of the hierarchical structure of the network is thus an excellent tool for identification of highly connected communities of nodes that are less densely connected with nodes belonging to other parts of the graph. Data partitioning is then performed by splitting the graph into clusters according to its community structure (Fig. 1C).

The resulting clusters of reads, representing different families of repetitive elements, can be further analyzed to gain information about the abundance and sequence composition of these repeats in the genome. Since the number of sequence reads generated by the random sequencing approach is proportional to the genomic abundance of their corresponding sequences, the cluster sizes provide a direct measure of the repeat proportion in the genome. Moreover, various statistics can be used to assess the type and properties of repetitive elements by evaluating the graph topology of individual clusters. These statistics include *graph diameter*, defined as the longest *distance *between any two nodes, where *distance *is the shortest path between the vertices; *graph density*, which is the ratio of the number of edges to the number of all possible edges; and *maximal degree*, which is the maximal number of edges leading to a single vertex of the graph. We have found that high graph density and maximal degree values are indicative of short tandem repeats or satellites, and that graph diameter is proportional to the repetitive element length (see examples provided below).

In addition, cluster graphs can be inspected visually. We used the 3D version of the Fruchterman and Reingold algorithm [17,11] to determine the informative placement of the vertices in 3D-space. This algorithm minimizes edge crossing and brings the vertices connected by an edge (i.e. reads with similarities) near each other. The placing of vertices is also affected by the attraction along the edges, which is proportional to the edge weight. In our case edge weight was based on the similarity score. In order to interactively investigate such graph structures we developed *SeqGrapheR*, an R package that provides a simple graphical user interface for interactive visualization of sequence clusters using the GGObi program and the R package rggobi [18,19]. *SeqGrapheR *also enables the selection of groups of reads from a graph and simultaneous viewing of the graph layout, sequence assembly results and similarity searches (Additional file 1).

### Application of graph-based methods to global repeat analysis in *Glycine max *and *Pisum sativum*

The analyzed datasets consisted of whole-genome 454 sequence reads from *G. max *[7] and *P. sativum *[6], representing small and medium-sized plant genomes with haploid DNA contents of 1,115 Mb and 4,300 Mb, respectively. The average read length was similar in the two datasets (115 and 104 nucleotides, respectively); however, they differed in genome coverage, due to the higher read quantity and much smaller genome size, almost 10-fold higher in *G. max *(0.07 ×) than in *P. sativum *(0.0077 ×). The analysis was performed using a computational pipeline integrating sequence similarity searches with a set of result-parsing and sequence-manipulation tools implementing the graph-based methods outlined above.

In the set of 717,383 reads from *G. max*, detected sequence overlaps resulted in a graph of 378,287 nodes connected by 16,547,366 edges. No sequence overlaps meeting our criteria (90% or better similarity over at least 55% of the longer sequence length) were found for the remaining 339,096 reads (47.2%), which probably represented single- or low-copy genomic sequences. In *P. sativum*, 319,402 analyzed reads yielded a graph including 247,153 nodes connected by 3,772,440 edges, while 72,249 reads (22.6%) remained single. It should be noted that in spite of the higher genome coverage of the *G. max *dataset, there was a much larger proportion of single reads than in *P. sativum*, reflecting the smaller repeat content in the former species.

**Figure 2:**
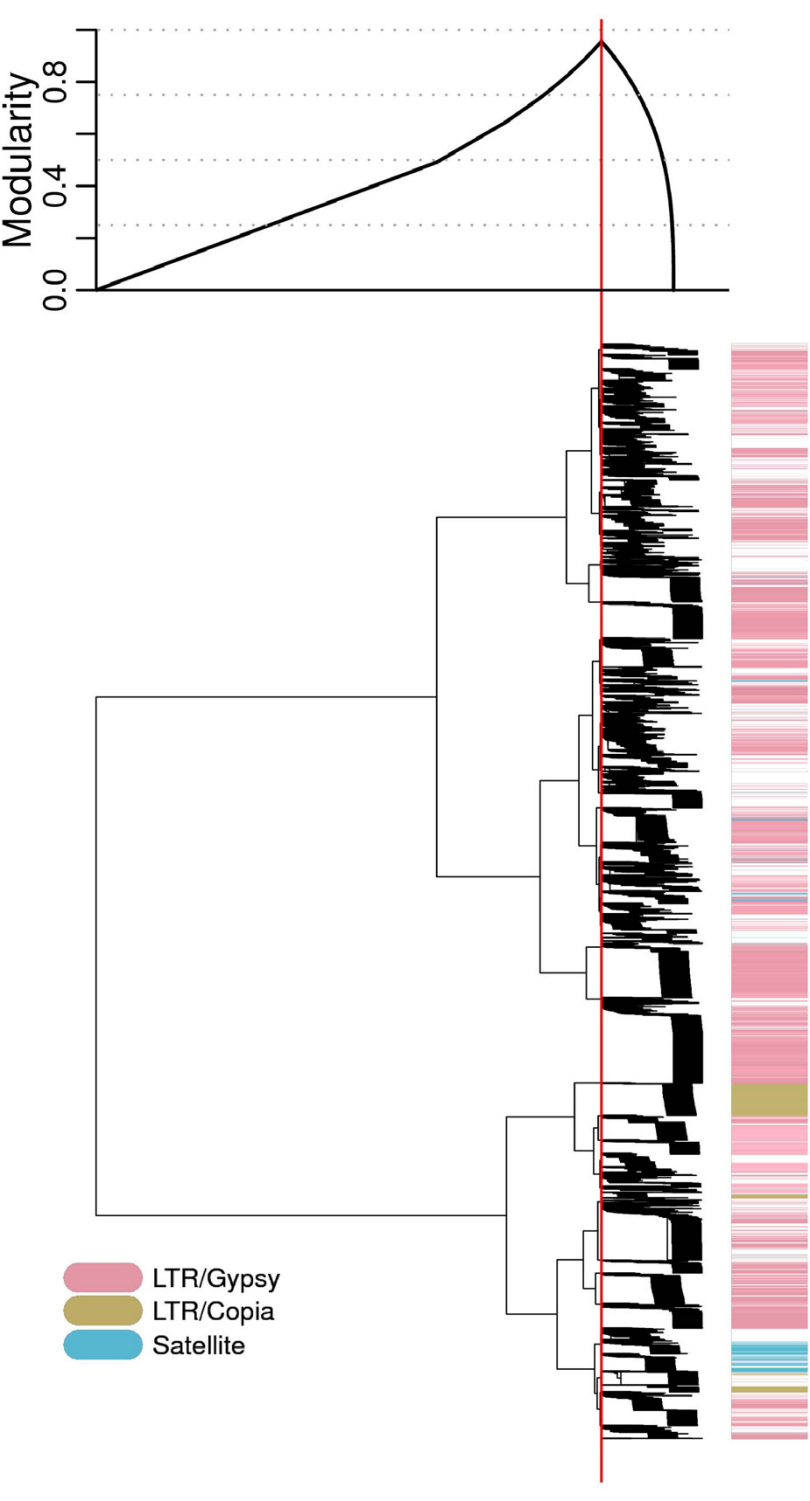
**Hierarchical organization of sequence reads**. Plot of modularity and dendrogram for the graph derived from 136,265 sequence reads of *P. sativum*. Each leaf of the tree represents a single sequence read (due to their high number it is not possible to distinguish individual reads). This tree corresponds to the largest connected component, which makes up 42% of all sequencing data. For each division of the hierarchical tree, the resulting modularity is shown above the dendrogram. The vertical red line represents the best division with maximal modularity, producing 230 subclusters. Repeats identified by the similarity search are shown on the colored vertical side bar, reads with no hits are left blank.

Using hierarchical agglomeration, reads included in the *G. max *and *P. sativum *graphs were partitioned into 63,992 and 20,549 clusters, respectively. When graphs were partitioned into individual connected components, the number of clusters obtained was slightly lower, at 63,761 (*G. max*) and 20,281 (*P. sativum*). This is because connected components can be either equivalent to hierarchical agglomeration clusters or can be broken down into multiple clusters (compare with Fig. 1A). This division into smaller clusters was the most frequent in large connected components and is demonstrated in Fig. 2, showing separation of the largest connected component of the *P. sativum *graph (136,265 reads) into clusters based on maximal modularity. To analyze the efficiency of clustering with respect to the separation of distinct repetitive elements, the reads within the clusters were scanned for similarity to a database of plant repetitive elements using RepeatMasker [20].The analysis showed that the largest connected component contained reads derived from several families of Ty3/gypsy and Ty1/copia elements and also from a tandem repeat. These distinct types of repetitive elements were separated by hierarchical agglomeration clustering. The dendrogram (Fig. 2) shows hierarchy of divisions of this connected component into 230 sub-clusters. In summary, the twelve largest connected components in *P. sativum*, which contained 158,628 reads, were further partitioned into 280 smaller clusters by hierarchical clustering. Similarly, in *G. max*, 16 connected components, accounting for 178,217 reads, were broken down into 242 clusters. On the other hand, 20,260 and 63,750 connected components that were composed of 88,225 and 200,070 reads were identical to hierarchical agglomeration clusters in *P. sativum *and *G. max*, respectively.

Comparison of our clustering analysis of *G. max *data with the previously published analysis produced by direct contig assembly [7] revealed that in both methods a similar fraction of reads was utilized in contigs or to form clusters. A total of 63,992 clusters consisted of 378,287 reads while 62,894 contigs comprised 384,339 reads. The major differences were found in the size and the number of large clusters and contigs consisting of more than 7 reads. Hierarchical agglomeration clustering resulted in a smaller number of larger clusters (2046 clusters with 110 reads on average) while partitioning by direct contig assembly led to higher number of smaller contigs (4,213 with 56 reads on average).

**Figure 3:**
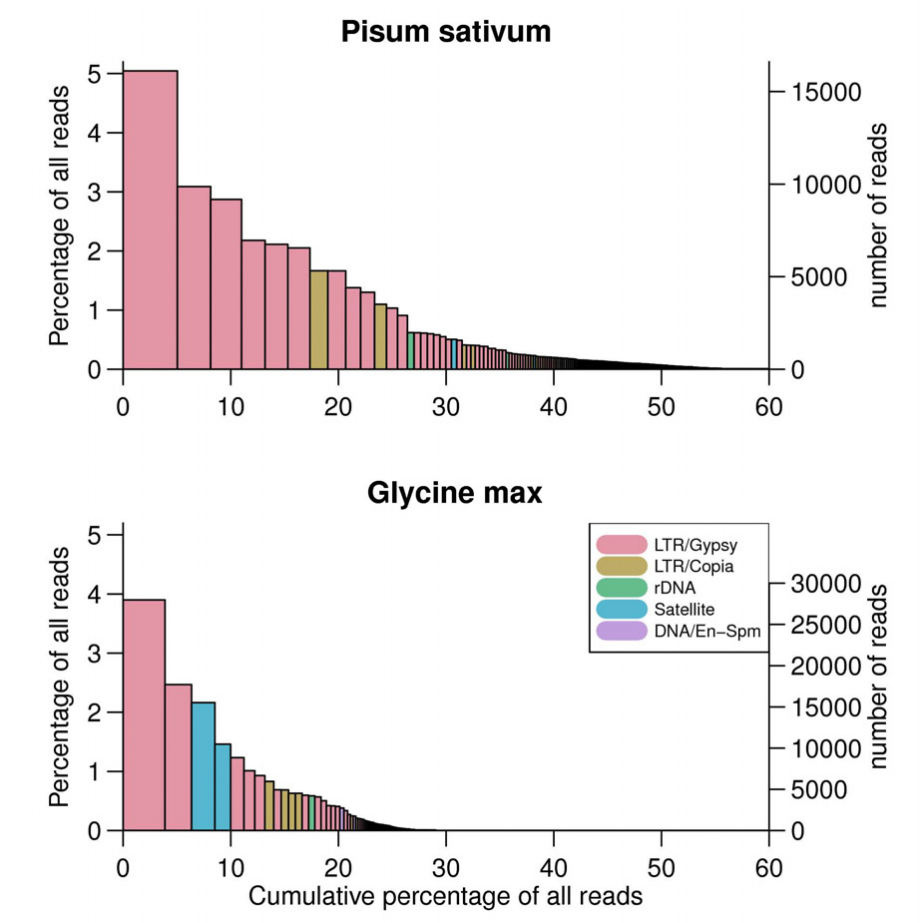
**Distribution of clusters in the genomes of *P. sativum *and *G. max *by size and class of repetitive element**. Histograms show the results of clustering based on the hierarchical agglomeration algorithm of all sequence reads. The height and width of the bars correspond to the number of reads in the cluster. The Y-axis shows both the percentage of the reads and number of reads in the clusters and the X-axis shows their cumulative content. Bars are colored according to the type of repeat present in the cluster, as determined by the similarity search.

The size and identity of the clusters summarized the known differences between genomes of *G. max *and *P. sativum *well (Fig. 3). Differences in the "slopes" of the bar plots reflect the distinct repetitive content in the *G. max *and *P. sativum *genomes. In general, the *P. sativum *genome can be described by a greater number of larger clusters than found in the *G. max *genome. Specifically, clusters that contain at least 0.1% of sequence reads are representative of 47% and 24% of the genome in *P. sativum *and *G. max*, respectively. The similarity search using RepeatMasker, which was performed for these largest clusters, enabled us to characterize the composition of these portions of the genomes (color coding in Fig. 3). Both genomes showed the greatest prevalence of Ty3/gypsy LTR elements and a smaller fraction of Ty1/copia elements. The *G. max *genome also contains highly amplified satellite sequences making up more than 3% of the genome, whereas *P. sativum *tandem repeats do not reach such high proportions.

### Detailed characterization of repeat families

**Figure 4:**
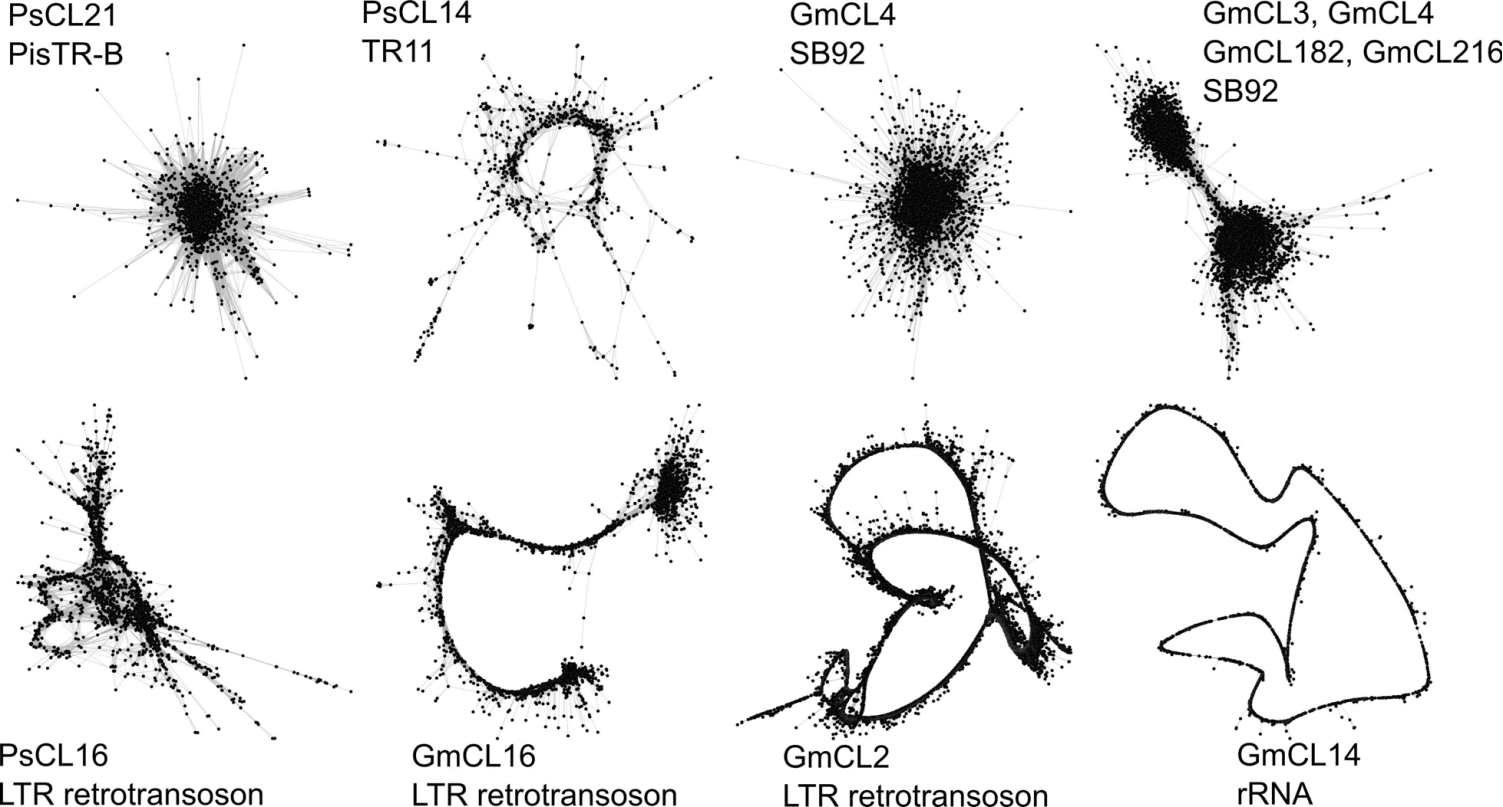
**Examples of graph layouts derived from clusters of repetitive sequences**. Graph layouts were calculated using the 3D version of Fruchterman and Reingold algorithm from which a 2D projection is shown. Individual reads are represented by vertices and similar reads are connected by edges. Individual clusters are described further in Table 1.

Examples of sequence clusters derived using hierarchical agglomeration are shown in Fig. 4 and their characteristics in Table 1 (see also Additional files 2 and 3 for detailed characteristics of a complete set of the 48 largest clusters). The first four clusters (Fig. 4, top row) are derived from various tandem repeats, including *P. sativum *satellites PisTR-B and TR-11 which make up 0.44% and 0.2% of the genome, respectively [6]. The different monomer lengths of these two satellites contribute to the distinct graph layouts. While PisTR-B, with a monomer length of 50 bp which is shorter than the average length of the sequence reads, forms a star-like structure, TR-11, with a monomer length five times larger than the average read length (510 bp), forms a graph with a ring-like shape. These same types of layouts were also observed in other clusters of tandem repeats. *G. max *satellite SB92, which occupies 3.7% of the *G. max *genome, was separated into four clusters, GmCL3, GmCL4, GmCL182 and GmCL216, accounting for 15,512, 10,474, 56 and 47 reads respectively. The layout of GmCL4 and the joint layout of all four SB92-containing clusters are shown in Fig. 4. The joint layout clearly shows two distinct major clusters in the SB92-like sequences, which were separated by the clustering algorithm. The minor clusters GmCL182 and GmCL216 cannot be visually distinguished from the major clusters GmCL3 and GmCL4 due to their manifold smaller size. SB92-like satellite sequences were also analyzed previously using direct contig assembly and similarity search [7]. This led to classification of the SB92-like sequences into 51 contigs. This again shows that direct contig assembly leads to a higher number of smaller contigs while hierarchical agglomeration can organize sequences more loosely into larger clusters. In general, the graphs based on satellites with short monomers are characterized by a high graph density and small diameter (Table 1). Conversely, graphs based on satellites with monomer lengths significantly longer than the read length are less dense and have a larger diameter (Fig. 4, PsCL21 and PsCL14).

**Table 1:** Characteristics of graph structures visualized in Fig. 4.

Cluster ID	Cluster size [reads]	Number of edges	Maximal degree	Graph diameter	Mean density	Maximal Modularity	Mean Blast score	Class of repetitive sequence	Name	Monomer length
PsCL21	1614	269813	1166	6	20.73%	0.19	103.84	Satellite	PisTR-B	50 bp
PsCL44	737	4536	81	19	1.67%	0.79	121.85	Satellite	TR-11	510 bp
GmCL4	10474	4859502	5293	7	8.86%	0.2	105.7	Satellite	SB92	92 bp
GmCL3, GmCL4, GmCL182, GmCL216	26089	11041274	6998	10	3.24%	0.50	101.72	Satellite	SB92	92 bp
PsCL16	1952	38347	200	19	2.01%	0.69	117.15	Ty3/gypsy	Ogre-PA	NA
GmCL16	3610	152029	455	34	2.33%	0.65	114.48	Ty3/gypsy	NA	NA
GmCL2	17701	959759	434	62	0.61%	0.7	126.53	Ty3/gypsy	NA	NA
GmCL14	4209	93866	85	91	1.06%	0.79	155.2	rDNA	rDNA	~7.6 kbp

**Figure 5:**
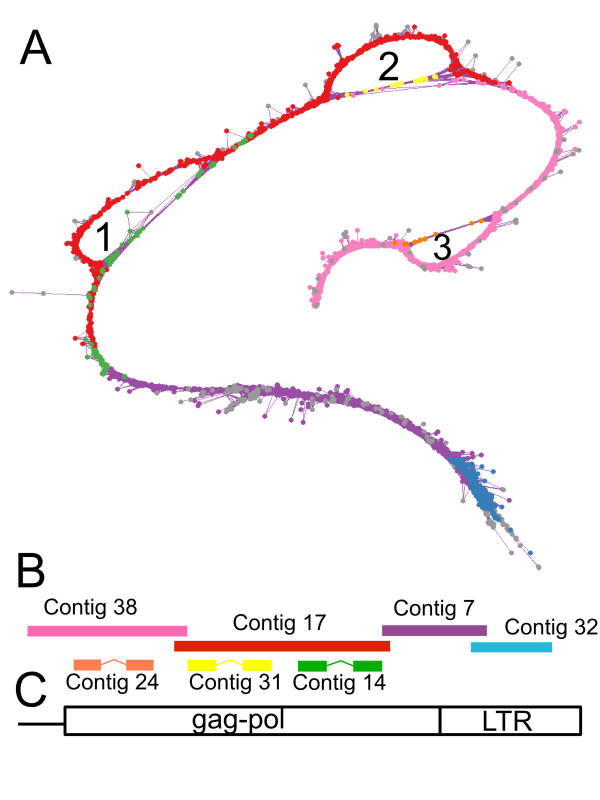
**Visualization of contigs in the *P. sativum *cluster PsCL7 using *SeqGrapheR***. (A) The graph layout was calculated using a 3D version of the Fruchterman and Reingold algorithm. The colors of the nodes are based on the results of the sequence assembly into contigs using the CAP3 program, which are then schematically represented in panel B. Numbers label the loops discussed in the text. The contig alignment is shown below the graph together with a diagram of corresponding regions of the Angela LTR-retrotransposon (C).

**Figure 6:**
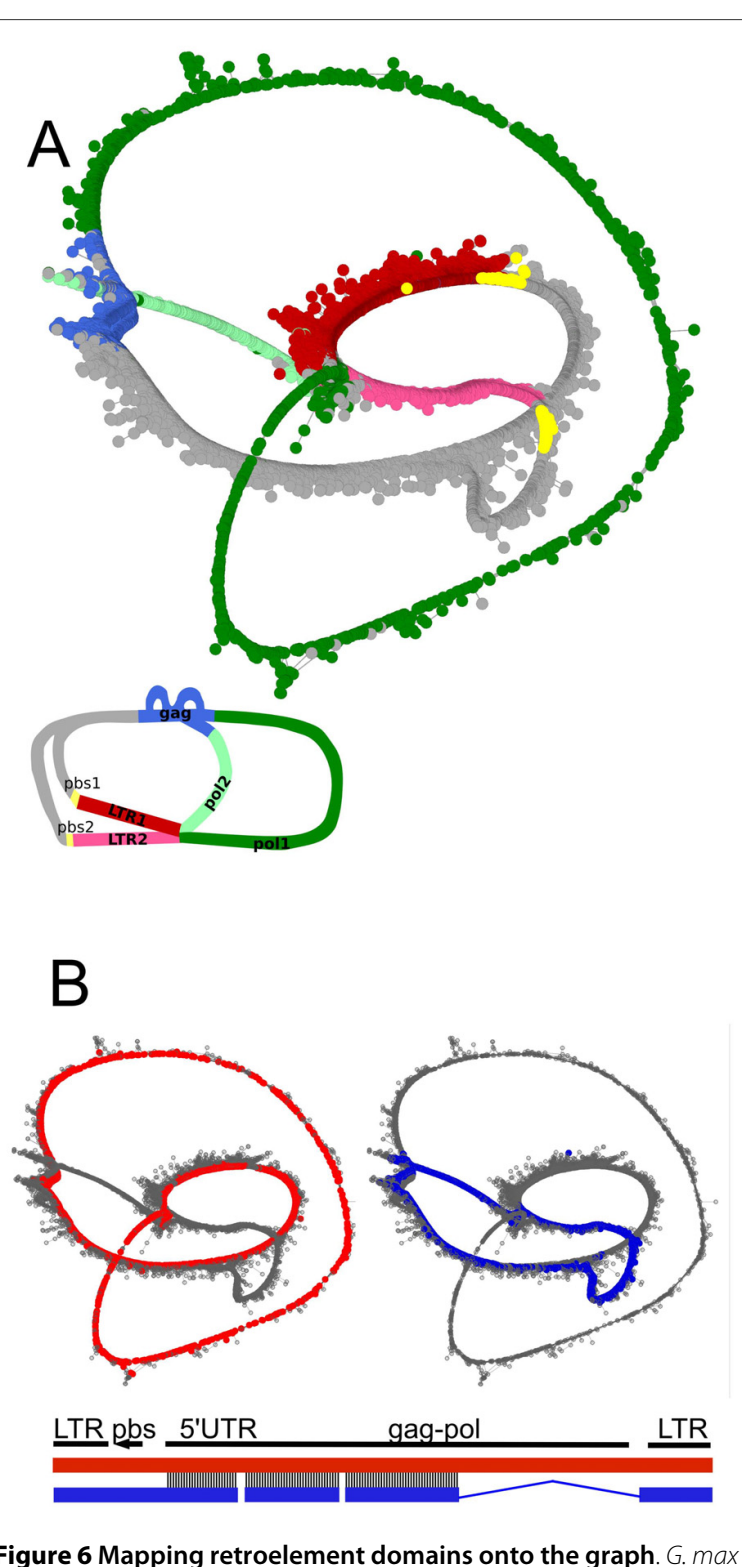
**Mapping retroelement domains onto the graph**. *G. max *clusters GmCL2 and GmCL35 representing the LTR-retrotransposon gmGYPSY10 were combined in one graph and visualized by *SeqGrapheR*. (A) Coloring of the nodes is based on the similarity search using blastx against our protein database, revealing the *gag *and *pol *coding domains, and the blastn search to detect primer binding sites (pbs). The scheme on the bottom left shows a simplified two-dimensional representation of the same graph. Different shades of red and green are used to distinguish between alternative sequences. To simplify the complicated structure of the layout, the graph has been modified for the purpose of better perceptible visualization by manually removing 214 nodes. (B) Correlation of the graph structure with genomic sequences. The coloring of the graphs is based on the similarity to elements identified in the genomic sequences AC235175 79155-70675 (red) and AC235457 19033-13657 (blue). A diagram of the alignment of these retroelements is shown below the graph in the same color. Vertical lines in the alignment show the regions with similarity.

Graphs derived from dispersed repeats with long units, like LTR retrotransposons, are characterized by the presence of long multiple parallel paths that form the more or less linearly organized parts of the graph layout (Fig. 4, graph PsCL16, GmCL16, GmCL2). The linear parts of the graph that contain nodes densely connected into thread-like structures represent potential contigs, which could be assembled from the reads in the cluster. This demonstrates an important feature of organizing reads using the graph, which is not limited by the stringent criteria required for contig assembly. Thus a group of reads that would normally be separated into distinct contigs can be captured in one graph due to the possibility of thread branching and looping (Fig. 4, GmCL2, Fig. 5). The rDNA cluster is the last example shown in Fig. 4 (GmCL14). The conservation of the rDNA copies in the genome (note the high mean Blast similarity score in Table 1) and its tandem organization is responsible for the tight circular layout. In general, a circular layout of a graph is a sign of either tandem organization or the presence of terminal repeats as in the case of LTR-retrotransposons (Fig. 4, GmCL2). Conversely, linearization of such a layout can be caused by the absence of sequencing coverage or by the presence of variable sequence regions with low coverage, which will cause partitioning into multiple clusters (see also detailed examples in Figs. 5 and 6). Overall, the contrasting graph layouts of distinct classes of repeats and their basic graph characteristics show that graph-based partitioning and graph based visualization of genomic 454 reads can serve well for the first coarse, unbiased characterization of sequence reads.

### Examples of analysis of individual sequence clusters

In this section we will demonstrate two examples of how the graph layouts explored using the *SeqGrapheR *tool are useful in elucidating the variability of repetitive elements and how they can help in reconstructing repetitive element consensus sequences. The first example is the pea cluster PsCL7 containing 5320 reads, representing the Ty1/copia LTR-retrotransposon Angela [21]. This seventh largest cluster identified in the *P. sativum *genome represents approximately 2% of all reads. Our attempts to assemble the sequences using the CAP3 program resulted in several imperfectly overlapping contigs. Even though it was not possible to assemble sequences of the cluster PsCL7 into a single contig, graph visualization using *SeqGrapheR *(Fig. 5A) showed one major thread of similar reads with three 'shortcuts'. A major thread of reads is representative of the most frequent form of the Angela elements while the shortcuts, containing fewer reads, correspond to less frequent form(s) with deletions (Fig. 5B). The concurrent view of the contig information and the graph layout is especially useful since it can be used in the manual assembly and finishing of a consensus sequence. In addition, *SeqGrapheR *enables counting of the reads present in individual structural parts of the graph and thus provides rapid quantification of element variation. By counting reads in the loops labeled 1, 2 and 3 (Fig. 5A) we estimated that there are 13%, 7% and 3% of Angela elements with deletions in these regions, respectively.

The second example shows the clusters GmCL2 and GmCL35 from *G. max *with 17,701 and 862 reads respectively. Since both these clusters are linked together by 61 similar reads and show similarity to the same LTR retrotransposon gmGYPSY10 [22], we combined the respective data into one graph structure with 18,563 reads that correspond to 2.6% of the *G. max *genome. The branched structure of the graph suggests that the retrotransposon family includes related elements that share some similar segments but differ in others. To further decipher the structure of the graph we marked all reads that were found by similarity search to be part of *gag *and *pol *regions. Moreover, similarity searching against a database of 3' ends of tRNA sequences also identified reads containing putative primer binding sites (pbs). Annotation of the graph using these features allowed us to estimate the positions of other structural regions, such as the LTRs (region upstream of the pbs and downstream of *pol*) and 5'UTR (region between pbs and *gag*). Since we could identify branching and two parallel threads corresponding to the LTRs, we concluded that the graph reflects the presence of two types of closely related elements (Fig. 6). Moreover, there are additional branch points in the graph, suggesting that the elements differ not only in the LTRs but also in other segments, namely parts of the 5'UTR and *pol*. Finally, a significant difference between the lengths of the two threads corresponding to *pol *suggests that one of the elements has a deletion in this region. To verify these conclusions, we investigated whether elements with the predicted structure really exist in the soybean genome. The full-length elements were retrieved from the GenBank nr database [23] using similarity searching against sequences from distinct parts of the graph. Comparison of two identified elements (AC235175 79155-70675 and AC235457 19033-13657) with sequences in the graph showed that our prediction, based solely on the graph structure and its annotation, was correct because both elements could be fully mapped and marked all threads visible in the graph (Fig. 6B).

## Discussion

Graph theory and methods are used in multiple areas of biology including phylogenetic analysis, the study of protein-protein interaction networks, and the description of gene regulatory networks [24]. In genomics, graph methods are used by several programs to facilitate sequence assembly [25-27]. In this paper, we have introduced a graph representation of sequence similarities of 454 sequence reads as a novel approach for the detection and characterization of repetitive sequences in eukaryotic genomes. We have implemented graph-based methods in a two-step analysis procedure, consisting of partitioning the data into clusters of overlapping reads representing individual repeated elements, and further characterization of these clusters.

The newly implemented hierarchical agglomeration algorithm is superior to the previously used methods and can be considered a balanced approach between the direct contig assembly [7,28] and clustering using the connected component method [6]. Compared to using contig sequences obtained by direct assembly of the whole set of non-partitioned 454 reads [7,29], the cluster-based approach is in principle more suitable for repeat detection and quantification as it better captures the sequence variability that is the typical feature of repetitive elements. Although the direct contig assembly approach can also be considered a special case of read clustering, its typical outcome is an excess of small clusters (i.e. contigs) consisting of reads with high similarities and a large number of singlets that are not assigned to any contig despite their partial similarity with other sequences. This is because direct assembly can only group sequences that can be organized into linear contigs without branching and excludes regions with low or no similarities. In this view, clustering can be considered a higher order classification, while contigs describe the smaller, more conserved parts of repetitive elements. Clustering can thus be used as a complementary method to sequence assembly, which can organize resulting contigs and singlets into logical higher order groups. The hierarchical agglomeration clustering was successful in the separation of groups of unrelated sequences (Fig. 2) and thus improved the main drawback of the connected component-based clustering, which suffers from the occasional formation of chimeric clusters [6]. In addition, it provided the possibility of investigating relationships between separate clusters by inspection of the hierarchical tree. It should be noted, however, that the hierarchical tree shown in Fig. 2 does not necessarily represent the phylogenetic relationship between individual clusters. Instead, it reflects the number of mutually similar reads between the clusters. Clusters in neighboring branches are thus those that contain a fraction of sequences with high similarities but in which the rest of the sequences is dissimilar. As a consequence, neighboring branches on the tree may contain related repetitive elements but also elements that share short regions of similarity but are otherwise unrelated. This can be explained either by common evolutionary origins of repetitive elements in these clusters or by co-localization of distinct repetitive elements in the genome either side by side or by insertion of one element into another. Thus, the hierarchical clustering analysis could be useful in investigating repeat co-localization in the genome caused by e.g. the insertional preferences of some mobile elements.

Ideally, every cluster would contain all reads from a particular class or type of repetitive element. While this is true for some repeats and clusters, we have found that some types of elements are separated into multiple clusters. This is either caused by a "missing link" where the chain of overlapping reads is interrupted, or by a "weak link" when the number of overlapping reads is low and the element is split into two or more subclusters. Low coverage or missing links can be caused by a number of factors. One possible explanation is a low read depth of the particular sequence, which increases the probability of gaps in the coverage. Another cause could be the presence of regions with high sequence variation and subsequent absence of similarity hits. The clustering outcome can also be affected by the total genome coverage of the sequencing itself. Since the coverage of a particular sequence is proportional to its abundance and to the total genome coverage, decrease in the amount of reads will cause breakage of less abundant repeats into multiple clusters due to insufficient coverage. On the other hand, clusters derived from more abundant repeats will remain unaffected by lower read coverage. With increasing read depth, clustering will be more efficient even for less frequent repeats.

In extreme instances where coverage is close to or even greater than 100% of the genome, the majority of the reads will be connected to one large connected component, which makes the connected component method useless. On the other hand, the modularity measure, which is used to quantify the quality of partitioning of the graph into clusters, is not only derived from the total number of nodes but also from the proportion of expected and observed edges inside and outside clusters. Consequently, clustering by hierarchical agglomeration should still be successful, even with very high sequencing coverage, since this partitioning is an outcome of the proportions of the edges in a cluster and not solely of their absolute number.

The second step of our analysis is characterization of the graph structure of sequence clusters by calculating various graph parameters and also by direct graph visualization. Sequence cluster visualization alone provides a fast and intuitive understanding of the relationship between reads and can reveal sequence variability and important structural features. Analysis of the graph layout can be extended by coloring the vertices based on sequence similarity with known elements or protein domains. Another application is the concurrent viewing of graph layouts and results from contig assembly using our interactive visualization tool *SeqGrapheR*, which helps significantly with manual finishing of the consensus sequence assembly. For example, in the Staden package for sequence assembly finishing dot-plotting is used as a tool to help decide which two contigs will be merged together [30]. While this approach is sufficient in principle, we suggest that a suitable graph layout view provides a more intuitive overview of the contig relationships than the dot plot view.

As the technology advances, new versions of 454 sequencing will provide larger quantities of longer reads than were used analysis described here. According to our preliminary tests, an increased read number and length will not negatively affect the outcome of our analysis. It should be noted, however, that analysis of large graphs is a computationally demanding task. For example, the running time for the Fruchterman-Reingold algorithm is proportional to *V*^2 ^+ *E *(*V *is the number of vertices, *E *is the number of edges) and this can be significant in the case of large clusters. On our computer which has 16 GB of RAM with eight 2 Ghz AMD processors, the single-threaded layout calculation takes from seconds up to hours depending on the cluster size and density. Based on our empirical testing, datasets containing 2.5 million reads with an average length of 400 nt can be processed when a swap partition with sufficient space is available. A potential limitation to our approach could be the presence of large proportions of simple tandem repeats, which can significantly slow down computation due to the frequent mutual similarities of their sequence reads. Hypothetically, if there are 100,000 reads derived from a tandem repeat with a monomer size smaller than the average read length, its corresponding graph structure could contain, in an extreme instance, billions of edges, because every read is similar to all reads derived from the same repeat. This can cause an increase both in the amount of data that has to be handled and in computation time. We suggest that including a prior analysis of sequencing data to identify highly abundant short tandem repeats and then removing these sequences from the dataset will have a great effect on computational time without affecting the clustering outcome. Moreover, significant progress in graph analysis can be expected when the use of GPU and parallelization is implemented [31,32].

## Conclusions

Compared to the previously used approaches for repeat characterization from 454 sequencing data, the graph-based method described in this work proved to be more precise in read clustering and superior in providing additional information about repeats in the investigated genomes. The hierarchical agglomeration algorithm used for clustering provides an additional level of classification information on top of contig assembly. This information can be used to assess the variability and evolutionary divergence of repeat families and to classify and characterize novel repetitive elements. Graph visualization of clusters proved to be useful for finishing consensus sequence assembly and identifying sequence variants of repetitive elements.

## Methods

The analyzes were performed on a computer cluster consisting of 32 CPUs and 2TB RAID running under the Debian Linux operating system. Parallelization of some tasks was performed using the Portable Batch System as implemented in TORQUE, an open source resource manager [33]. The computational pipeline consisted of a set of scripts written in BioPerl [34] and R [12,10] and also utilized some programs (tgicl, mgblast, cap3) included in the TGICL package [9]. Sequencing data were preprocessed to remove identical reads, which are technical artifacts of the 454 technology. The remaining reads were then searched for mutual similarities using mgblast with the following parameters: -p 85 -W18 -UT -X40 -KT -JF -F "m D" -v90000000 -b90000000 -D4 -C80 -H 30. The program output was parsed to select pairs of reads with similarities greater than 90% over at least 55% of the longer sequence length.

The information about read similarities that passed the specified threshold was processed using the R script *fgclust *to build and further analyze a graph where the reads were represented by vertices and their similarities by edges connecting the overlapping reads. Clustering analysis was performed using a hierarchical agglomeration algorithm [13] for detecting *communities *[14] which are defined as groups of vertices in a graph that are more densely connected internally than with the rest of the graph. To find the optimal graph partitioning into these communities, the greedy algorithm implemented in the open source software package *igraph *[11] was used. In principle, in a randomly connected graph *G(n, p) *with *n *vertices, the probability *p *of an edge existing between two vertices *i *and *j *is defined as

$$p=\frac{k_{i}k_{j}}{2m},$$

where *k*_*i *_and *k*_*j *_are degrees of vertices *i *and *j*, respectively, and *m *is the total number of edges in the graph. The presence of communities within the graph results in deviations from the random distribution of edges and can be evaluated using *modularity *(*Q*). The modularity quantifies quality of the graph division into communities and is defined as [15]:

$$Q=\frac{1}{2m}\sum_{ij}\left[A_{ij}-\frac{k_{i}k_{j}}{2m}\right]\delta (c_{x},c_{y})$$

where *A*_*ij *_is an element of the adjacency matrix of the graph (if vertices are connected, *A*_*ij *_= 1, otherwise *A*_*ij *_= 0). Vertices *i *and *j *belong to communities *c*_*x *_and *c*_*y*_, respectively, and the function *δ(x, y) *is 1 if *x *= *y *and 0 otherwise. Modularity *Q *then corresponds to the fraction of edges that fall within communities minus the corresponding value in the random graph. If the fraction of within community edges is not different from what is expected for the random graph, then *Q *is zero. Conversely, high *Q *values represent a good division of the graph into communities. In the case of our analysis, the random graph was modeled using the parameters (the number of vertices and edges, the degree of individual vertices, and the graph density) obtained from the graph derived from 454 sequencing data in order to make these two graphs comparable. To find division with the highest modularity, a greedy optimization of modularity *Q *was performed [11]. At the beginning of this procedure, each vertex of the graph forms a singleton community. Then, for each pair of communities, expected improvement of modularity when they merge is calculated. The community pair which gives the maximum *ΔQ *is joined into new community and the whole process is repeated and eventually leads to single community when there are no community pairs to merge. The optimal partitioning of the graph into communities is then represented by the community structure at the iteration with the highest modularity *Q*. As a result of this analysis, *fgclust *identifies the clusters and produces a list of read content of the clusters, a postscript file with a graphical representation of the clusters, files containing pre-calculated graph layouts, and a file with information about basic graph characteristics. Another R program, *SeqGrapheR *package, was then used for 18 interactive exploration of the sequence clusters. These scripts are available as Additional file 4 and will also be deposited at a dedicated web page http://w3lamc.umbr.cas.cz/lamc/resources.php. Sequence assembly was performed using the cap3 program with the -O '-p 80 -o 40' parameters specifying overlap percent identity and length cutoff, respectively.

Similarity searches of sequence reads to known repetitive elements were performed using RepeatMasker [20] and its Viridiplantae database augmented with our own custom-made database of repeats from selected plant species. Additional searches were performed using blastn and blastx [35] against GenBank nr [23] and our own protein database.

## Authors' contributions

PNo and JM developed the software for the analysis pipeline. PNe analyzed the retrotransposon data. JM initiated and guided the project. All authors contributed to the preparation of the manuscript and approved its final version.

## Supplementary Material

Additional file 1 **An example of cluster visualization using SeqGrapheR program**. A screenshot of the SeqGrapheR interactive graphical user interface demonstrating various functions of the program. Click here for file

Additional file 2 **The largest sequence clusters identified in *Pisum sativum***. A list of 48 largest clusters showing their characteristics, graph layouts, and assignment to repeat families. Click here for file

Additional file 3 **The largest sequence clusters identified in *Glycine max***. A list of 48 largest clusters showing their characteristics, graph layouts, and assignment to repeat families. Click here for file

Additional file 4 **Software tools for the graph-based characterization of repetitive sequences**. A file archive including the R script *fgclust *for partitioning of sequence reads, the R package *SeqGrapheR *for interactive visualization of graphs derived from clusters of sequence reads, installation instructions and user manual. Click here for file
