# Supplementary material for: Graph-based clustering and characterization of repetitive sequences in next-generation sequencing data
Source: BMC Bioinformatics. 2010 Jul 15;11:378. doi: 10.1186/1471-2105-11-378 (PMC2912890; doi:10.1186/1471-2105-11-378)
Supplement: Additional file 1 — An example of cluster visualization using SeqGrapheR program. A screenshot of the SeqGrapheR interactive graphical user interface demonstrating various functions of the program. [file 1471-2105-11-378-S1.PDF]

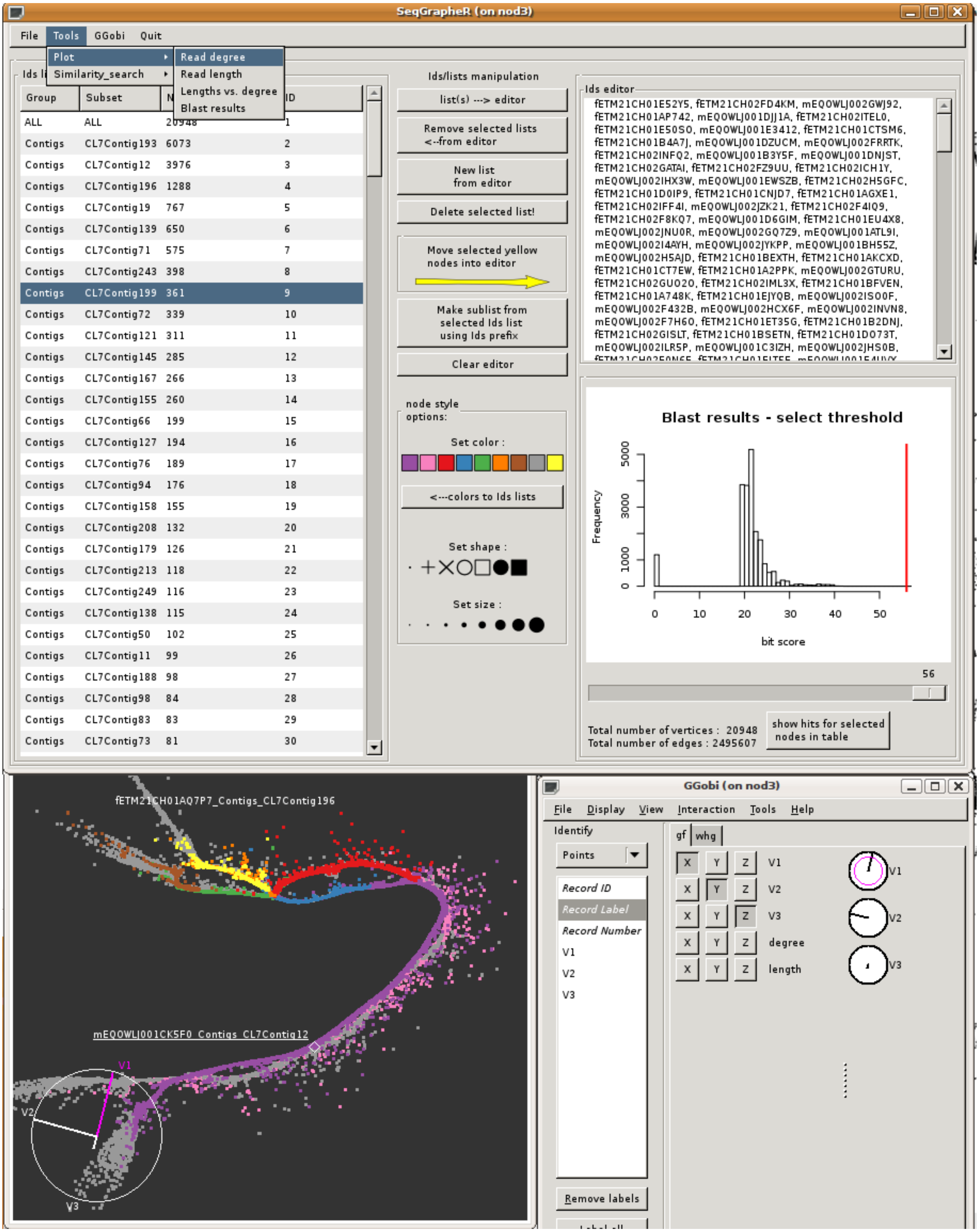

Supplemental figure 1. Screenshot from *SeqGrapheR* program. *SeqGrapheR* is interactive graphical user interface which integrates GGobi visualization tool with Graph based on DNA sequence information, DNA assembly results and similarity searches.
