# Supplementary material for: Graph-based clustering and characterization of repetitive sequences in next-generation sequencing data
Source: BMC Bioinformatics. 2010 Jul 15;11:378. doi: 10.1186/1471-2105-11-378 (PMC2912890; doi:10.1186/1471-2105-11-378)
Supplement: Additional file 2 — The largest sequence clusters identified in Pisum sativum. A list of 48 largest clusters showing their characteristics, graph layouts, and assignment to repeat families. [file 1471-2105-11-378-S2.PDF]

Pisum sativum sequence clusters layout and their characteristics

Cluster	CL1	CL2	CL3	CL4
RepeatMasker best hit	LTR/gypsy/Ogre	LTR/gypsy/Ogre	LTR/gypsy/Ogre	LTR/gypsy/Ogre
Number of reads	16117	9871	9173	6960
Number of pairs	463914	300081	221020	177907
Maximal degree	292	349	219	324
Diameter	116	35	46	46
Mean density	0.004	0.006	0.005	0.007
Modularity	0.84	0.77	0.82	0.75
Mean Blast score	113.44	121.18	112.03	115.72
	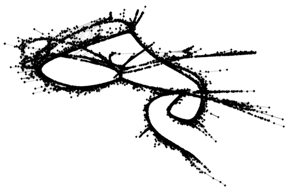	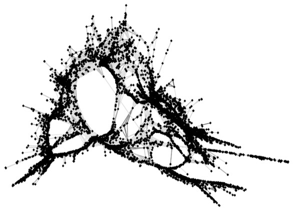	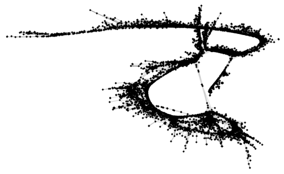	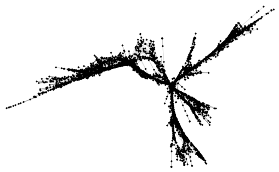
Cluster	CL5	CL6	CL7	CL8
RepeatMasker best hit	LTR/gypsy/Ogre	LTR/gypsy/peabody	LTR/copia/Angela	LTR/gypsy/Ogre
Number of reads	6747	6553	5320	5313
Number of pairs	211582	207384	273351	97459
Maximal degree	258	186	254	203
Diameter	59	104	79	67
Mean density	0.009	0.010	0.019	0.007
Modularity	0.75	0.83	0.67	0.85
Mean Blast score	116.04	125.48	123.13	116.81
	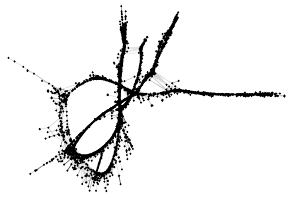	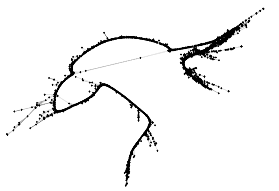	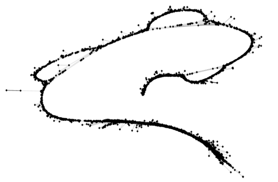	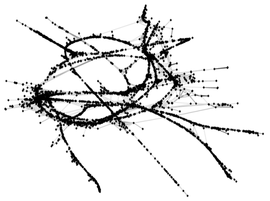
Cluster	CL9	CL10	CL11	CL12
RepeatMasker best hit	LTR/gypsy/Ogre	LTR/gypsy/Ogre	LTR/copia/SIRE	LTR/gypsy/Ogre
Number of reads	4407	4160	3507	3302
Number of pairs	178231	73717	58569	29989
Maximal degree	281	280	133	81
Diameter	26	32	46	37
Mean density	0.018	0.009	0.010	0.006
Modularity	0.67	0.76	0.8	0.89
Mean Blast score	132.8	112.64	119.57	113.92
	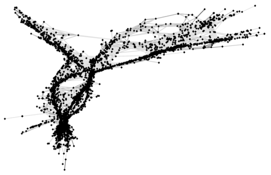	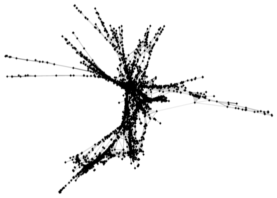	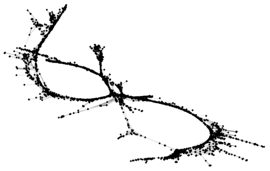	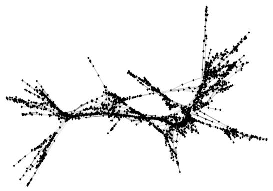

Cluster	CL13	CL14	CL15	CL16
RepeatMasker best hit	LTR/gypsy/Ogre	rDNA	LTR/gypsy/Ogre-PA	LTR/gypsy/Ogre-PA
Number of reads	2909	1980	1970	1952
Number of pairs	103019	33390	11031	38347
Maximal degree	291	58	58	200
Diameter	28	123	38	19
Mean density	0.024	0.017	0.006	0.020
Modularity	0.61	0.77	0.87	0.69
Mean Blast score	126.33	160.34	108.85	117.15
	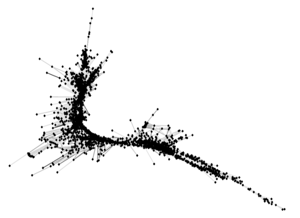	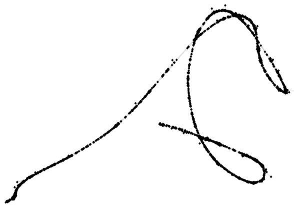	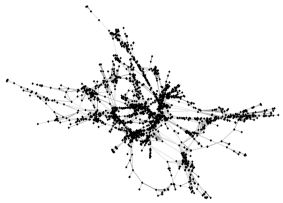	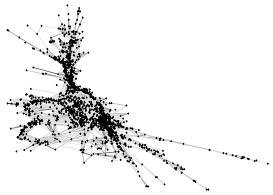

Cluster	CL17	CL18	CL19	CL20
RepeatMasker best hit	LTR/Gypsy	LTR/gypsy/Ogre-PA	LTR/gypsy/Ogre	LTR/gypsy/Ogre
Number of reads	1914	1855	1756	1618
Number of pairs	57088	15983	28847	31408
Maximal degree	166	74	176	173
Diameter	39	35	39	22
Mean density	0.031	0.009	0.019	0.024
Modularity	0.72	0.85	0.74	0.6
Mean Blast score	134.22	115.21	110.82	134.7
	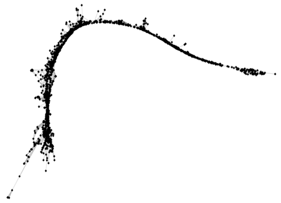	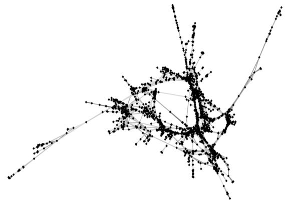	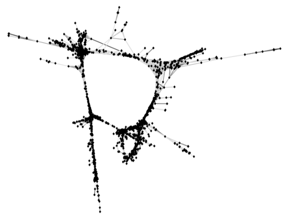	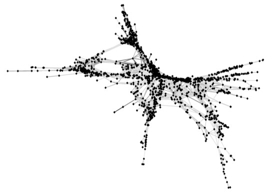

Cluster	CL21	CL22	CL23	CL24
RepeatMasker best hit	Satellite/PisTR-B	LTR/gypsy/Ogre-PA	LTR/Copia	LTR/gypsy/Ogre-PA
Number of reads	1614	1566	1310	1297
Number of pairs	269813	17123	20250	12757
Maximal degree	1166	83	78	75
Diameter	6	38	46	20
Mean density	0.207	0.014	0.024	0.015
Modularity	0.19	0.74	0.75	0.84
Mean Blast score	103.84	109.41	124.61	111.1
	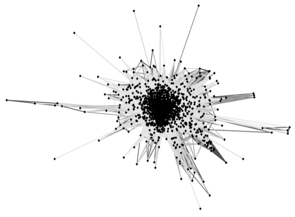	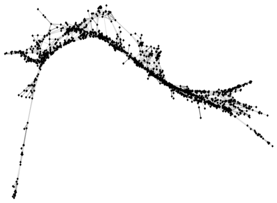	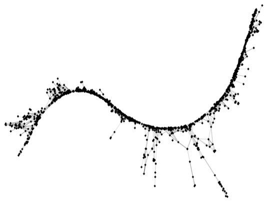	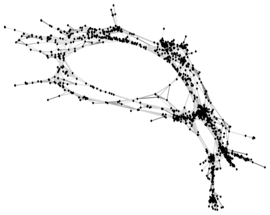

Cluster	CL25	CL26	CL27	CL28
RepeatMasker best hit	LTR/Copia	LTR/gypsy/Ogre	LTR/gypsy/Ogre	N/A
Number of reads	1288	1282	1238	1235
Number of pairs	32145	13745	12327	16582
Maximal degree	111	92	73	187
Diameter	41	22	32	20
Mean density	0.039	0.017	0.016	0.022
Modularity	0.64	0.82	0.85	0.55
Mean Blast score	128.04	117.04	114.11	106.32
	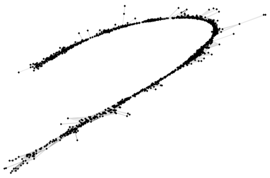	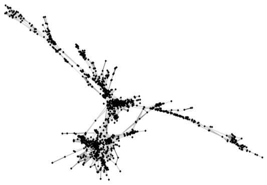	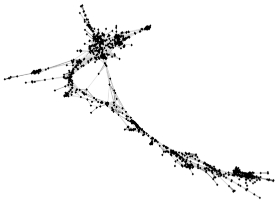	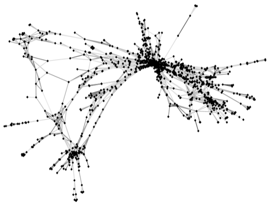

Cluster	CL29	CL30	CL31	CL32
RepeatMasker best hit	LTR/Gypsy	LTR/gypsy/Ogre-PA	LTR/gypsy/Ogre-PA	LTR/gypsy/Ogre-PA
Number of reads	1125	1124	1039	1036
Number of pairs	15624	6180	10555	8635
Maximal degree	88	54	73	79
Diameter	32	37	25	21
Mean density	0.025	0.010	0.020	0.016
Modularity	0.74	0.84	0.75	0.76
Mean Blast score	110.6	115.62	108.43	112.33
	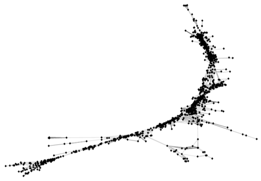	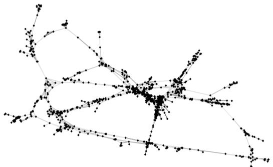	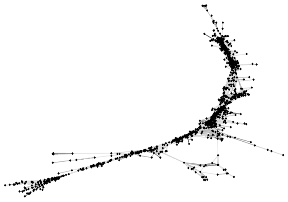	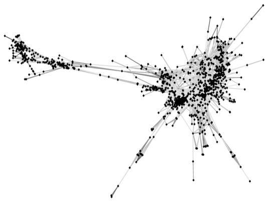

Cluster	CL33	CL34	CL35	CL36
RepeatMasker best hit	LTR/gypsy/peabody	rDNA	LTR/gypsy/Ogre	LTR/gypsy/Ogre
Number of reads	1027	899	876	835
Number of pairs	15594	47723	2926	7772
Maximal degree	123	253	27	84
Diameter	28	17	43	36
Mean density	0.030	0.118	0.008	0.022
Modularity	0.69	0.46	0.92	0.73
Mean Blast score	128.63	135.86	109.46	117.43
	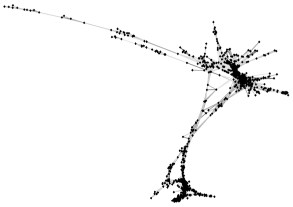	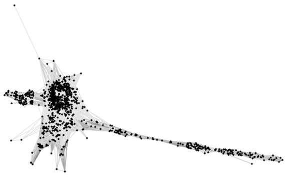	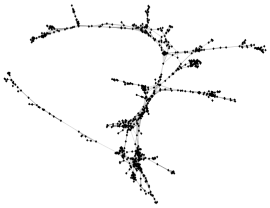	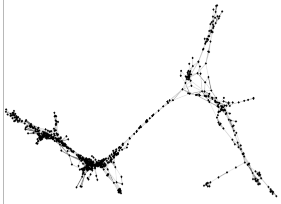

Cluster	CL37	CL38	CL39	CL40
RepeatMasker best hit	N/A	LTR/Copia	LTR/gypsy	LTR/Gypsy
Number of reads	822	806	795	792
Number of pairs	7790	12944	9081	8690
Maximal degree	55	133	67	58
Diameter	46	24	35	37
Mean density	0.023	0.040	0.029	0.028
Modularity	0.8	0.54	0.76	0.82
Mean Blast score	141.4	132.14	139.87	114.46
	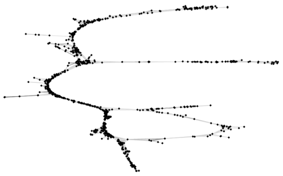	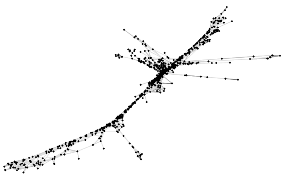	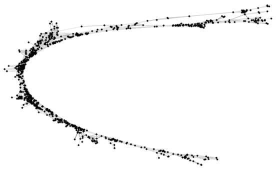	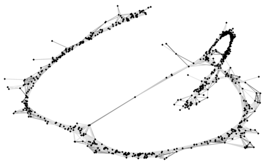

Cluster	CL41	CL42	CL43	CL44
RepeatMasker best hit	LTR/Copia	LTR/gypsy/Ogre	LTR/Copia	Satellite/TR11
Number of reads	763	760	739	737
Number of pairs	6088	5754	7776	4536
Maximal degree	72	63	60	81
Diameter	42	40	39	19
Mean density	0.021	0.020	0.029	0.017
Modularity	0.74	0.81	0.73	0.79
Mean Blast score	122.74	118.15	123.15	121.85
	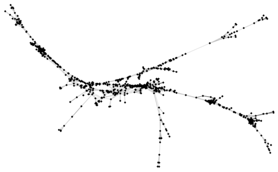	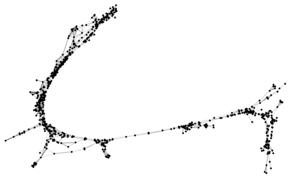	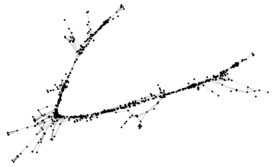	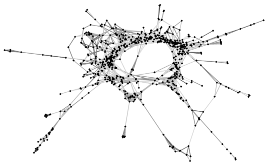

Cluster	CL45	CL46	CL47	CL48
RepeatMasker best hit	LTR/copia/SIRE	LTR/gypsy	LTR/gypsy/Ogre	LTR/copia/SIRE
Number of reads	695	684	672	669
Number of pairs	4973	4475	6420	4218
Maximal degree	46	35	112	34
Diameter	38	63	25	54
Mean density	0.021	0.019	0.028	0.019
Modularity	0.87	0.89	0.61	0.88
Mean Blast score	121.49	125.85	127.91	119.43
	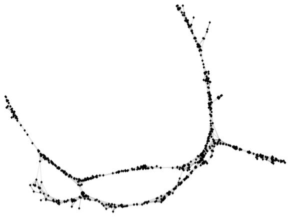	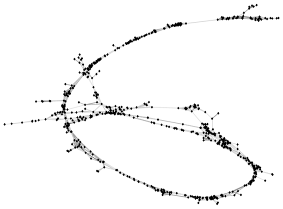	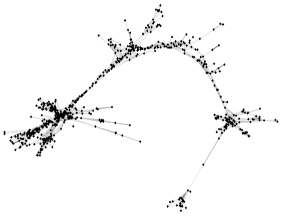	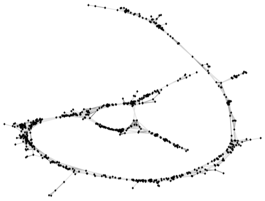
