# Supplementary material for: Graph-based clustering and characterization of repetitive sequences in next-generation sequencing data
Source: BMC Bioinformatics. 2010 Jul 15;11:378. doi: 10.1186/1471-2105-11-378 (PMC2912890; doi:10.1186/1471-2105-11-378)
Supplement: Additional file 3 — The largest sequence clusters identified in Glycine max. A list of 48 largest clusters showing their characteristics, graph layouts, and assignment to repeat families. [file 1471-2105-11-378-S3.PDF]

Glycine max sequence clusters layout and their characteristics

Cluster	CL1	CL2	CL3	CL4
RepeatMasker best hit	LTR/Gypsy/Tat-STR	LTR/Gypsy	Satellite/SB92	Satellite/SB92
Number of reads	27975	17701	15512	10474
Number of pairs	1244355	959759	6177243	4859502
Maximal degree	540	434	6998	5293
Diameter	112	62	7	7
Mean density	0.003	0.006	0.051	0.089
Modularity	0.71	0.7	0.24	0.2
Mean Blast score	134.64	126.53	98.61	105.7
	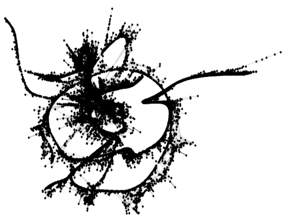	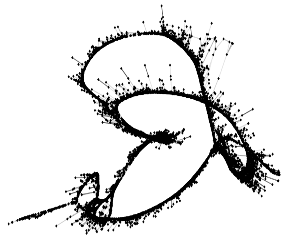	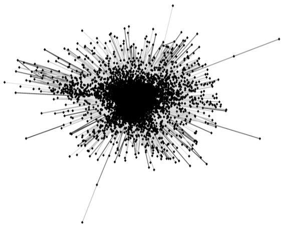	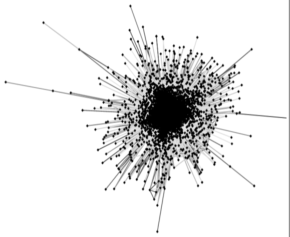
Cluster	CL5	CL6	CL7	CL8
RepeatMasker best hit	LTR/Gypsy/Tat-STR	LTR/Gypsy	LTR/Gypsy/Tat-STR	LTR/Copia
Number of reads	8852	7264	6676	5953
Number of pairs	206214	101863	1015359	186279
Maximal degree	190	91	1293	197
Diameter	47	179	22	163
Mean density	0.005	0.004	0.046	0.011
Modularity	0.78	0.87	0.53	0.78
Mean Blast score	125.22	129.71	107.57	125.11
	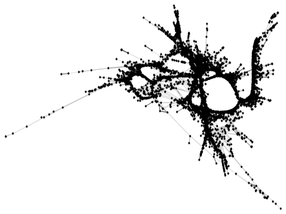	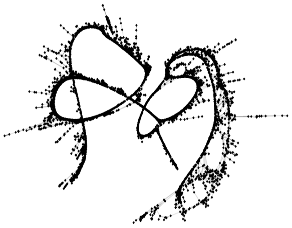	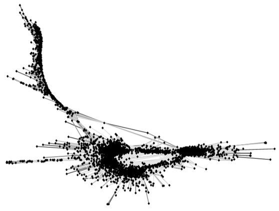	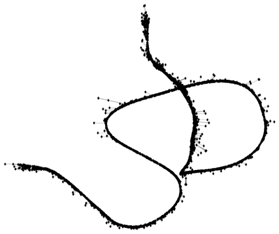
Cluster	CL9	CL10	CL11	CL12
RepeatMasker best hit	LTR/Gypsy	LTR/Copia	LTR/Copia	LTR/Copia
Number of reads	4942	4924	4519	4516
Number of pairs	142613	52502	103351	259989
Maximal degree	213	83	261	369
Diameter	49	121	27	25
Mean density	0.012	0.004	0.010	0.026
Modularity	0.73	0.88	0.66	0.59
Mean Blast score	128.83	125.78	122.8	131.13
	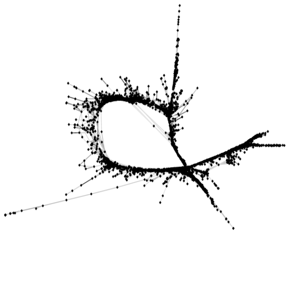	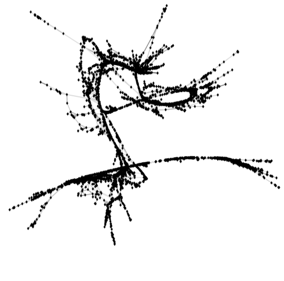	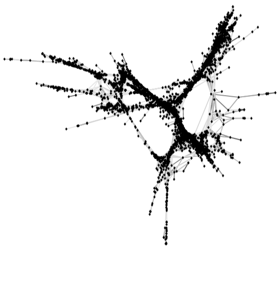	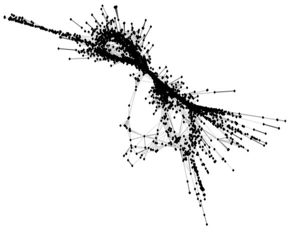

Cluster	CL13	CL14	CL15	CL16
RepeatMasker best hit	N/A	rDNA	LTR/Gypsy	LTR/Gypsy/Tat-STR
Number of reads	4286	4209	4081	3610
Number of pairs	43210	93866	74216	152029
Maximal degree	114	85	102	455
Diameter	60	91	59	34
Mean density	0.005	0.011	0.009	0.023
Modularity	0.83	0.79	0.79	0.65
Mean Blast score	115.71	155.2	127.09	114.48
	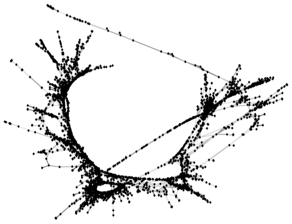	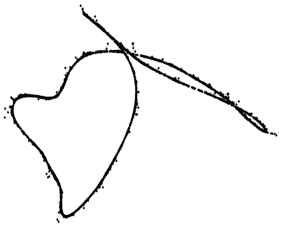	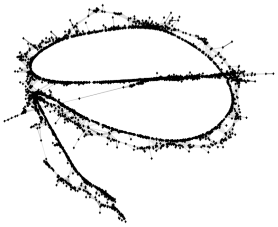	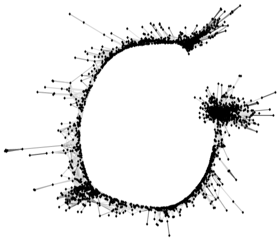

Cluster	CL17	CL18	CL19	CL20
RepeatMasker best hit	LTR/Gypsy	N/A	LTR/Gypsy/Tat-STR	DNA/En-Spm
Number of reads	3010	2993	2931	2728
Number of pairs	41757	44435	27244	22073
Maximal degree	74	247	72	59
Diameter	77	11	80	119
Mean density	0.009	0.010	0.006	0.006
Modularity	0.83	0.7	0.87	0.89
Mean Blast score	119.52	123.23	123.44	116.01
	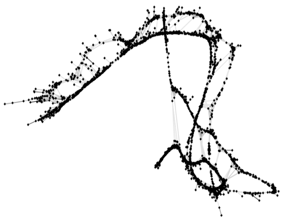	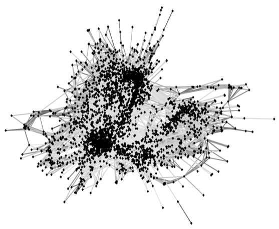	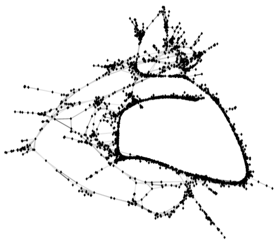	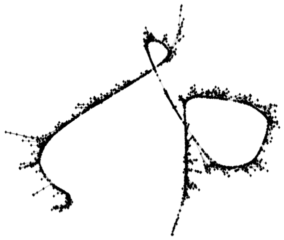

Cluster	CL21	CL22	CL23	CL24
RepeatMasker best hit	LTR/Gypsy/Tat-STR	N/A	N/A	DNA/En-Spm
Number of reads	2416	1951	1785	1748
Number of pairs	62975	12150	20459	13957
Maximal degree	199	48	100	86
Diameter	24	78	41	63
Mean density	0.022	0.006	0.013	0.009
Modularity	0.64	0.89	0.75	0.82
Mean Blast score	119.84	118.74	113.31	126.11
	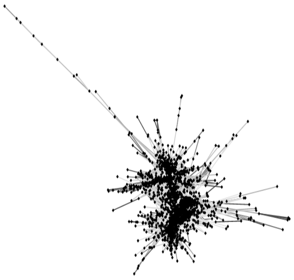	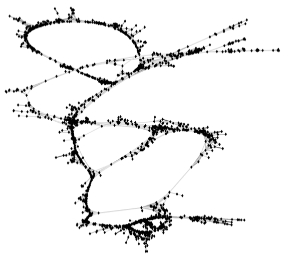	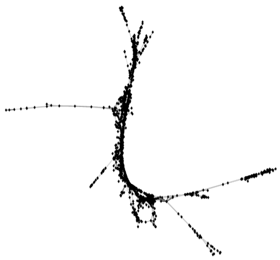	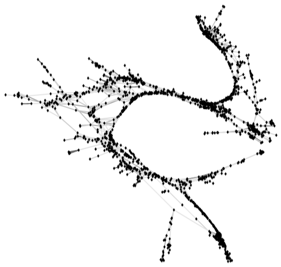

Cluster	CL25	CL26	CL27	CL28
RepeatMasker best hit	LTR/Copia	LTR/Copia	LTR/Gypsy/Tat-STR	LTR/Gypsy/Tat-STR
Number of reads	1501	1446	1396	1330
Number of pairs	22569	18256	14478	101627
Maximal degree	80	87	86	414
Diameter	62	43	42	10
Mean density	0.020	0.017	0.015	0.115
Modularity	0.77	0.77	0.75	0.35
Mean Blast score	119.17	126.27	126.67	116.74
	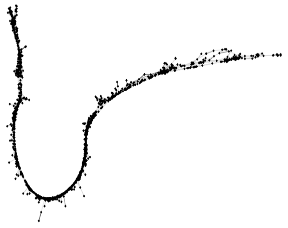	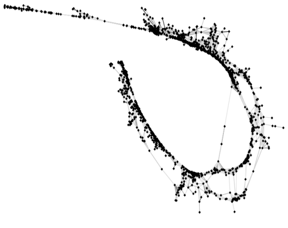	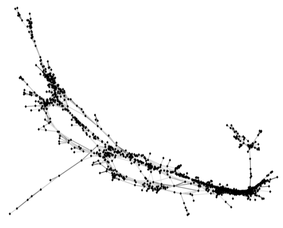	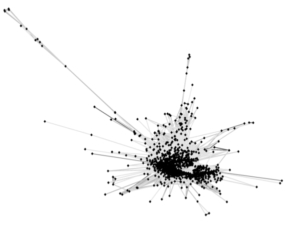

Cluster	CL29	CL30	CL31	CL32
RepeatMasker best hit	N/A	LTR/Copia	LTR/Gypsy	LTR/Gypsy
Number of reads	1190	1159	1090	1040
Number of pairs	6317	6424	18754	6515
Maximal degree	57	41	123	65
Diameter	31	59	32	32
Mean density	0.009	0.010	0.032	0.012
Modularity	0.86	0.9	0.68	0.76
Mean Blast score	119.16	120.46	128.25	122.41
	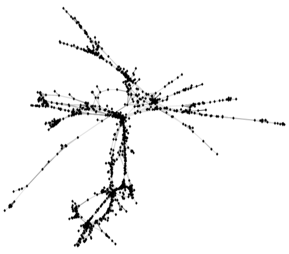	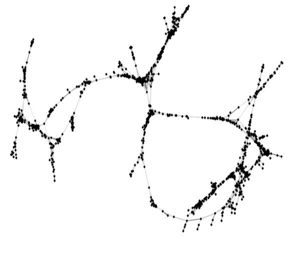	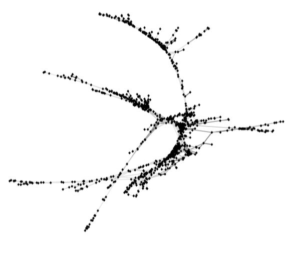	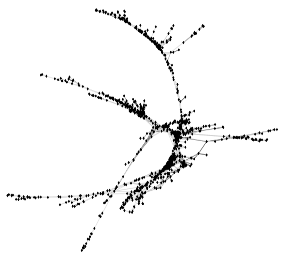

Cluster	CL33	CL34	CL35	CL36
RepeatMasker best hit	LTR/Gypsy/Tat-STR	N/A	LTR/Gypsy	LTR/Gypsy/Tat-STR
Number of reads	1007	962	862	850
Number of pairs	9038	9569	9717	8028
Maximal degree	62	62	53	54
Diameter	64	29	48	38
Mean density	0.018	0.021	0.026	0.022
Modularity	0.86	0.75	0.82	0.83
Mean Blast score	123.91	124.06	130.07	129.63
	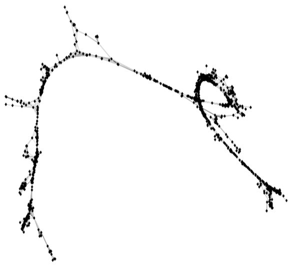	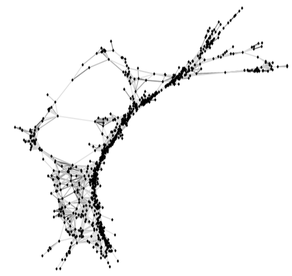	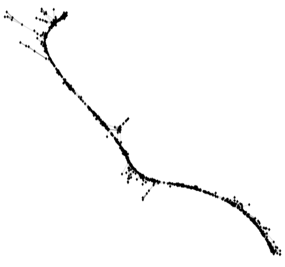	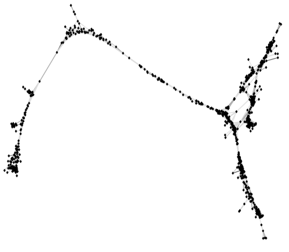

Cluster	CL37	CL38	CL39	CL40
RepeatMasker best hit	LTR/Gypsy/Tat-STR	LTR/Copia	N/A	N/A
Number of reads	801	767	757	732
Number of pairs	8223	2820	3153	12284
Maximal degree	70	24	28	67
Diameter	42	63	46	35
Mean density	0.026	0.010	0.011	0.046
Modularity	0.78	0.92	0.89	0.65
Mean Blast score	129.66	125.88	121.02	141.81
	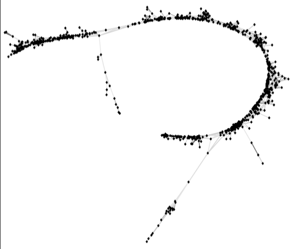	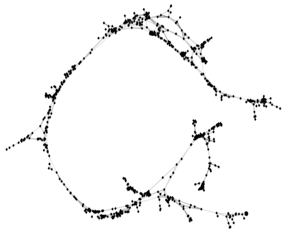	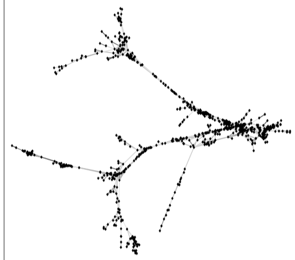	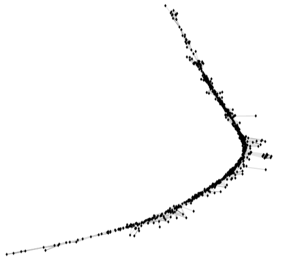

Cluster	CL41	CL42	CL43	CL44
RepeatMasker best hit	LTR/Gypsy/Tat-STR	LTR/Copia	N/A	LTR/Gypsy/Tat-STR
Number of reads	724	720	664	658
Number of pairs	4311	5962	10579	8076
Maximal degree	39	45	99	132
Diameter	55	48	21	21
Mean density	0.016	0.023	0.048	0.037
Modularity	0.83	0.79	0.56	0.54
Mean Blast score	123.1	125.35	131.67	135.17
	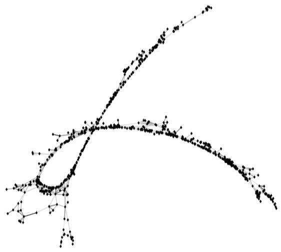	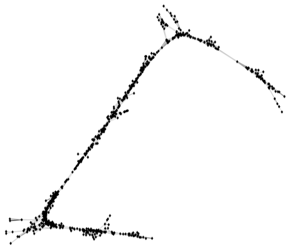	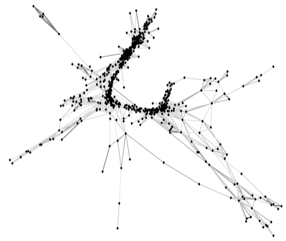	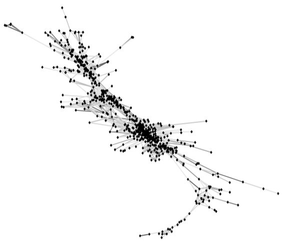

Cluster	CL45	CL46	CL47	CL48
RepeatMasker best hit	LINE/RTE-BovB	LTR/Gypsy	N/A	N/A
Number of reads	654	616	605	572
Number of pairs	8691	5333	2470	2376
Maximal degree	51	59	27	34
Diameter	48	44	48	71
Mean density	0.041	0.028	0.014	0.015
Modularity	0.74	0.73	0.89	0.9
Mean Blast score	142.42	127.59	125.7	130
	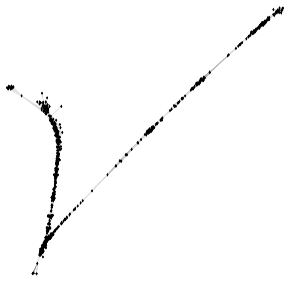	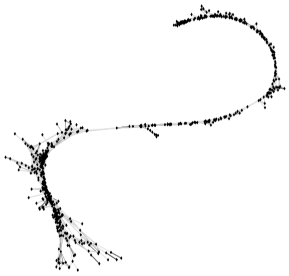	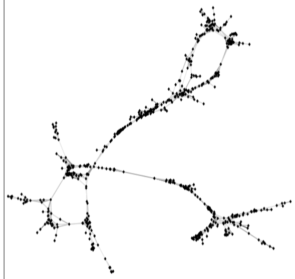	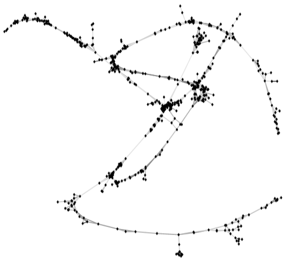
