# Supplementary material for: Graph-based clustering and characterization of repetitive sequences in next-generation sequencing data
Source: BMC Bioinformatics. 2010 Jul 15;11:378. doi: 10.1186/1471-2105-11-378 (PMC2912890; doi:10.1186/1471-2105-11-378)
Supplement: Additional file 4 — Software tools for the graph-based characterization of repetitive sequences. A file archive including the R script fgclust for partitioning of sequence reads, the R package SeqGrapheR for interactive visualization of graphs derived from clusters of sequence reads, installation instructions and user manual. [file 1471-2105-11-378-S4.GZ › programs/SeqGrapheR_manual.pdf]

SeqGrapheR

Petr Novak
Email:petr@umbr.cas.cz

May 10, 2010

Abstract

The SeqGrapheR package provides a simple GUI for R using rggobi, RGtk2 and gWidgets toolkits for the visualization of sequence read clusters as a graph layout. This approach is decribed in [REF] To display graphs interactively, SeqGrapheR uses the GGobi visualization program.

Contents

1 Installing SeqGrapheR 1

2 Starting SeqGrapheR 1

3 Ids list selector 3

4 Manipulation with Ids lists 4

5 Histogram area 5

6 Similarity search 6

7 GGobi window 6

8 Project 7

1 Installing SeqGrapheR

SeqGrapheR requires installation of the program GGobi (www.ggobi.org) and for full functionality it is necessary to install NCBI blast with programs blastall and megablast in the path. NCBI blast can be obtained from
`ftp.ncbi.nlm.nih.gov/blast/executables/LATEST/`. The R packages gWidget, Rgtk2, igraph, rggobi, gWidgetsRGtk2 and cairoDevice can be installed from CRAN with the command as

```
> install.packages('igraph', dep=TRUE)
```

Additionally, the required package Biostrings can be obtained from Bioconductor website (`www.bioconductor.org`). To install the package start R and enter:

```
>source("http://bioconductor.org/biocLite.R")
>biocLite("Biostrings")
```

When all required libraries are installed in R, SeqGrapheR can be installed with command:

```
>install.packages('SeqGrapheR_0.4.1.tar.gz',repos=NULL)
```

OS compatibility: Linux -recommended, Windows will require the installation of gtk libraries, functionality in MacOS was not tested.

2 Starting SeqGrapheR

To run SeqGrapheR GUI start R and enter:

```
>library(SeqGrapheR)
>SeqGrapheR()
```

It is possible to issue the SeqGrapheR() command multiple times and run independent instances of GUI. To start with, a graph must be imported first from the SeqGrapheR main window (Fig. 1). Currently there are four possibilities of how to import a graph. First, the graph can be imported from GL format. This file is generated during clustering processes by a fgclust script. GL format is actually an external representation of an R object GL where GL$G is an igraph object and GL$L is a matrix with layout coordinates calculated by the Fruchterman-Reingold algorithm. The second supported format is the ncol format. The .ncol edge file is a simple 2 (or 3) columns file where two vertices are on each line separated by tabulator:

```
vertex1name    vertex2name    [optionalWeight]
vertex1name    vertex3name    [optionalWeight]
```

When the .ncol file is loaded, the graph layout is calculated. This can take some time, from minutes for graphs with hundreds of vertices to couple of hours from graphs with 20,000 vertices. The progress of the layout calculation is show in the terminal. The third option is to load sequences in fasta format. In that case, megablast is run first for all-to-all comparison to find all edges of the graph, then the graph layout is calculated. The fourth option is to load a graph as the whole project. This option will also import additional project-associated information like lists of Ids, contig information, or results of a similarity search. When a graph is loaded, the histogram of degree should appear in the right bottom corner and the graph in the ggobi window will pop up. Also, the first Ids list is created from all sequence identifiers.

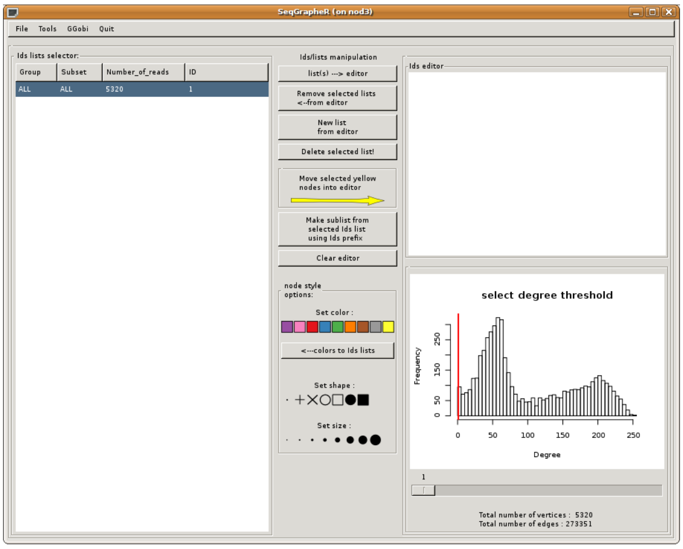

Figure 1: SeqGrapheR main window

The graph is shown in separate scatterplot window of GGobi program (Fig. 2). By default, only nodes without vertices are shown. Hiding the edges significantly speeds up the rendering and the manipulation of the graph (in extreme cases GGobi can crash during attempts to display all edges). To see or hide the edges, one can use options *Edges/Show lines only* or *Edges/hide edges* in the graph window. The initial graph scatterplot shows only the first two dimensions. It is possible to interactively change the point of view by switching into 3D from the main GGobi window using the

View/Rotation option. There are different modes of interaction with the graph which can be changed from main GGobi window. When 3D view is on (rotation) the possible interactions are *Rotation* - enable to change the point of view, *Brush* is used for the labeling group of nodes .*Identify* is used for identification of individual points (reads). More information can be found in the GGobi manual.Yellow color is used for selection. While it is possible to change the color scheme, it is not recommended for correct interaction of SeqGrapher and GGobi.

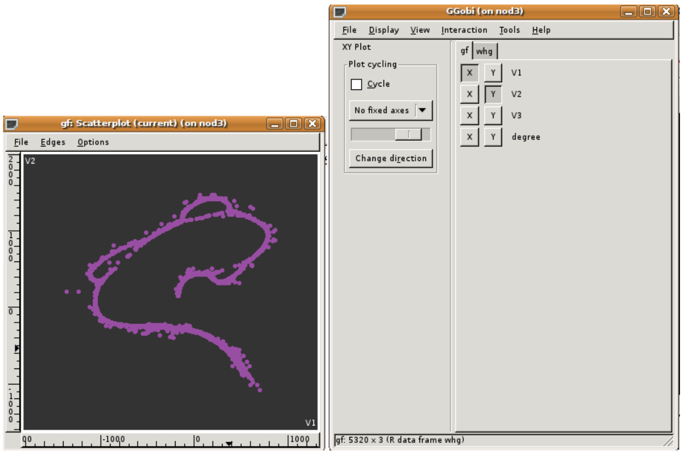

Figure 2: GGobi graph and main window

3 Ids list selector

Ids list selector is the table on the left side. This table contains all created or imported lists of sequence names (Ids). A list of Ids can be imported either from a file or by manual entry into the Ids editor. To import the list of Ids from a file, use the option *File/Import/Single Ids list to editor* which can upload a file with ids separated by spaces, tabs, or commas. Only valid Ids are imported. Another option is to import multiple Ids list by *File/Import/Multiple Ids list*. Multiple lists must be in format:

```
>groupname1 Subsetname1 Number_of_Ids1
Ids1 Ids2 Ids3 Ids4 Id5...
>groupname Subsetname Number_of_Ids
Ids12 Ids22 Ids32 Ids42 Id52...
```

It is possible to use an alternative format where each Id is associated with frequency; in this case every Id must be followed by the frequency (integer):

```
>groupname1 Subsetname1 Number_of_Ids1
Ids1 Freq1 Ids2 Freq2 Ids3 Freq3.....
>groupname2 Subsetname2 Number_of_Ids2
Ids21 Freq21 Ids22 Freq22 Ids23 Freq22.....
```

For a list with associated frequencies, an histogram of frequencies can be shown by selecting a single list in the Ids list selector and then using the option from the *Tools/Plot/Frequency of Ids* menu from selected list. To create an Ids list based on a contig assembly, an ACE file containing the sequence assembly information can be loaded. Multiple lists will be created, each list containing an Ids name for each contig. Every Ids lists can be marked by a different color/shape/size in the graph simply by selecting the row(s) with the Ids list and clicking on the *Node style options* to select the desired style.

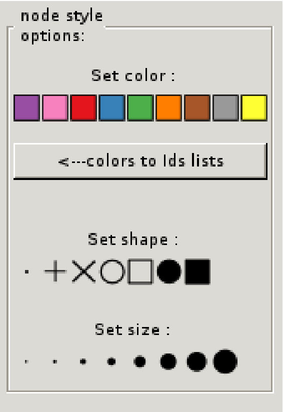

Figure 3: Node style options

4 Manipulation with Ids lists

Buttons in the upper middle panel can be used for creating and editing Ids lists. Ids can be moved from the Ids list table into the editor, edited manually, and multiple lists can be merged together.A list can also be created from the yellow nodes which were selected by a brushing, by using the histogram, or by another method.

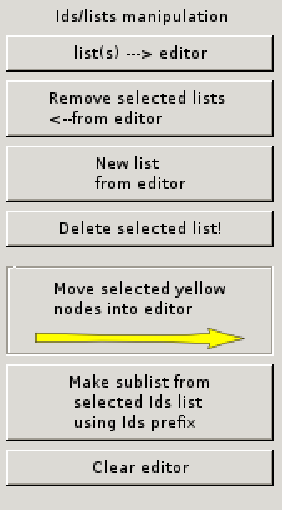

Figure 4: Ids list manipulation area

Additionally, multiple lists based on the color of the graph can be created by clicking the button below the strip color selector <—*colors to Ids list*. Sequence Ids can be also used for creating prefix-specific sublists. With selected Ids list, click on the *Make sublist from selected Ids list using Ids prefix*, then you will be asked for the prefix length. Multiple subsets will be created; each subset with unique N-letter long prefix Ids. This option can be used for comparative analysis when multiple sequence sources were pooled (e.g. from different species) and were labeled with a subset-specific prefix (Fig. 5).

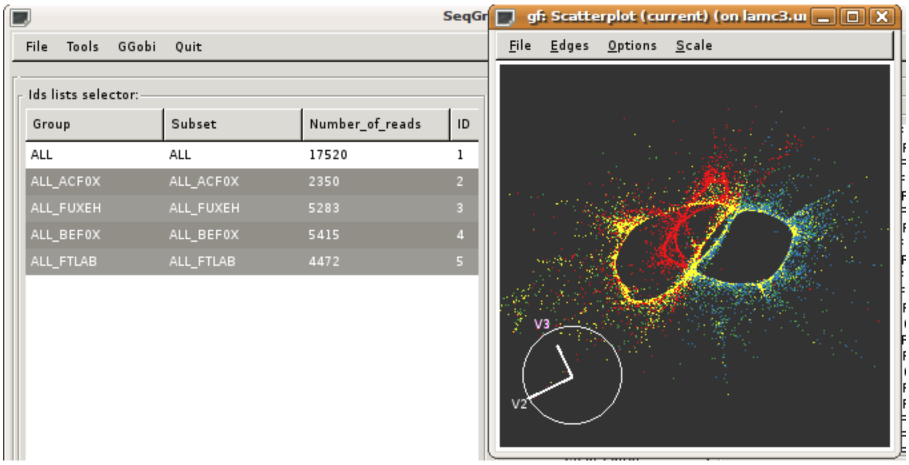

Figure 5: Example of subsets based on Ids prefixes. 5 letter long species-specific prefixes were used to create four sublists (labeled as ALL prefix) to distinguish the species-specific part of the graph.

5 Histogram area

When a graph is loaded, a histogram showing the distribution of all node degrees will appear on the bottom left. The degree of a node is the number of edges which ends at that node. In the case of a graph derived from sequence reads, nodes with higher degrees are those which are similar to more sequences while reads with lower degree have less similar counterparts.A region in the graph with a higher degree thus represents a sequence which is in the genome in more copies or is more conserved. To identify such regions in the graph, a slider below the histogram can be used to select the threshold for coloring. All nodes above the threshold will be labeled by a yellow color. The figure bellow shows the an example of the graph derived from a LTR-retrotransposon where nodes with degree greater than 120 (yellow) correspond to the sequences derived from LTRs which are present in the genome in more copies than internal sequences. When sequences are also loaded, a histogram of the read lengths can be shown in histogram ar ea as well. To switch between histogram views, use *Tools/Plot/Read degree* or *Tools/Plot/Read length*.

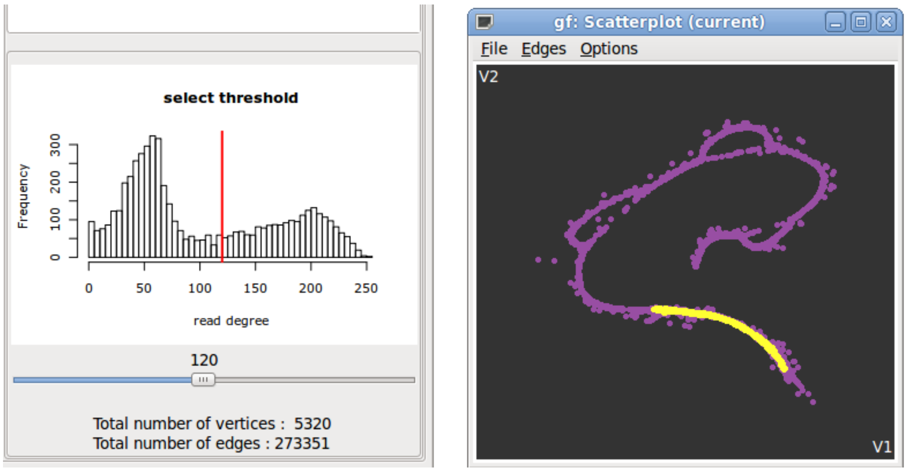

Figure 6: Example of selection of nodes with high degree using Histogram.

6 Similarity search

To use this option, NCBI blast must be installed (blastall 2.2.18 was used for SeqGrapheR testing). To run a similarity search, sequences corresponding to the graph nodes must be imported first. To search for similarity to proteins or DNA use *Tools/Similarity search/Protein blastx* or *Tools/Similarity search/DNA blastn*. The SeqGrapheR window will be non-reactive until the search is finished. Results are first shown in the histogram window which show a histogram of the blast similarity bit-scores. Histogram with the last blast results can be also re-opened by Tools/Plot/Blast results.

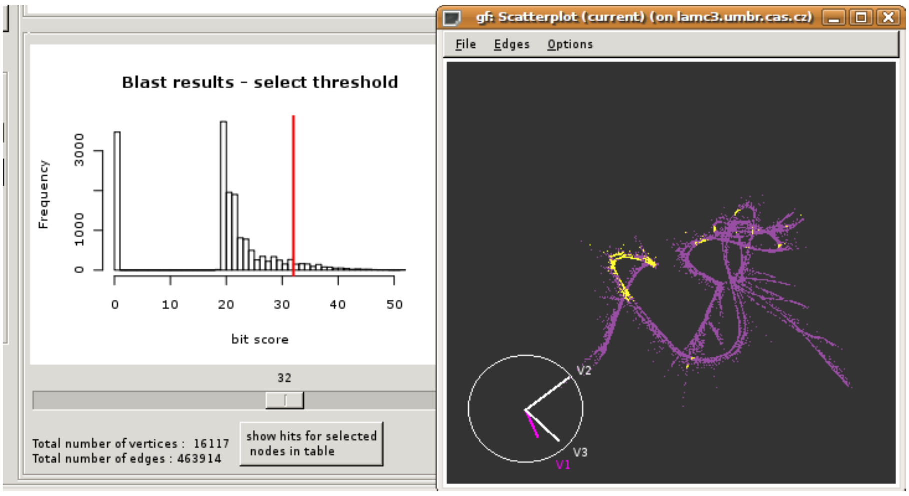

Figure 7: Example of Blast results shown as a histogram. All nodes with blast score greater or equal to the threshold (set here to 32) are highlighted.

To see the table with blast hit information, use the slider to select a bit score threshold and press *show hits for selected nodes* in the table button below the histogram. Blast results in the table can be sorted by clicking on the corresponding column header. Selected rows can be used for an Ids list creation by moving them into Ids editor (*move selected to Ids editor* button). Currently only the last similarity search is stored in the memory. When a new search is run, the previous results are overwritten. A table with the blast results can be exported or imported using *File/Export/Blast results* or *File/Import/Blast results (tabular format)*

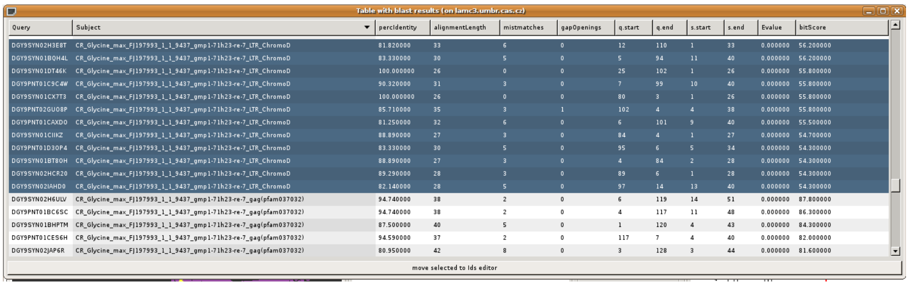
Table with blast results (on lamc3.umbr.cas.cz)

Query	Subject	percIdentity	alignmentLength	mistmatches	gapOpenings	q.start	q.end	s.start	s.end	Evalue	bitScore
DGY9SYN02H3E8T	CR_Glycine_max_FJ197993_1_1_9437_gmp1-71h23-re-7_LTR_ChromoD	81.820000	33	6	0	12	110	1	33	0.000000	56.200000
DGY9SYN01BQH4L	CR_Glycine_max_FJ197993_1_1_9437_gmp1-71h23-re-7_LTR_ChromoD	83.330000	30	5	0	5	94	11	40	0.000000	56.200000
DGY9SYN01DT46K	CR_Glycine_max_FJ197993_1_1_9437_gmp1-71h23-re-7_LTR_ChromoD	100.000000	26	0	0	25	102	1	26	0.000000	55.800000
DGY9PNT01C9C4W	CR_Glycine_max_FJ197993_1_1_9437_gmp1-71h23-re-7_LTR_ChromoD	90.320000	31	3	0	7	99	10	40	0.000000	55.800000
DGY9SYN01CX7T3	CR_Glycine_max_FJ197993_1_1_9437_gmp1-71h23-re-7_LTR_ChromoD	100.000000	26	0	0	80	3	1	26	0.000000	55.800000
DGY9PNT02GU08P	CR_Glycine_max_FJ197993_1_1_9437_gmp1-71h23-re-7_LTR_ChromoD	85.710000	35	3	1	102	4	4	38	0.000000	55.800000
DGY9PNT01CAXD0	CR_Glycine_max_FJ197993_1_1_9437_gmp1-71h23-re-7_LTR_ChromoD	81.250000	32	6	0	6	101	9	40	0.000000	55.500000
DGY9SYN01CIIKZ	CR_Glycine_max_FJ197993_1_1_9437_gmp1-71h23-re-7_LTR_ChromoD	88.890000	27	3	0	84	4	1	27	0.000000	54.700000
DGY9PNT01D3OP4	CR_Glycine_max_FJ197993_1_1_9437_gmp1-71h23-re-7_LTR_ChromoD	83.330000	30	5	0	95	6	5	34	0.000000	54.300000
DGY9SYN01BT8OH	CR_Glycine_max_FJ197993_1_1_9437_gmp1-71h23-re-7_LTR_ChromoD	88.890000	27	3	0	4	84	2	28	0.000000	54.300000
DGY9SYN02HCR20	CR_Glycine_max_FJ197993_1_1_9437_gmp1-71h23-re-7_LTR_ChromoD	89.290000	28	3	0	89	6	1	28	0.000000	54.300000
DGY9SYN02IAHD0	CR_Glycine_max_FJ197993_1_1_9437_gmp1-71h23-re-7_LTR_ChromoD	82.140000	28	5	0	97	14	13	40	0.000000	54.300000
DGY9SYN02H6ULV	CR_Glycine_max_FJ197993_1_1_9437_gmp1-71h23-re-7_gag(pfam037032)	94.740000	38	2	0	6	119	14	51	0.000000	87.800000
DGY9PNT01BC6SC	CR_Glycine_max_FJ197993_1_1_9437_gmp1-71h23-re-7_gag(pfam037032)	94.740000	38	2	0	4	117	11	48	0.000000	86.300000
DGY9SYN01BHPTM	CR_Glycine_max_FJ197993_1_1_9437_gmp1-71h23-re-7_gag(pfam037032)	87.500000	40	5	0	1	120	4	43	0.000000	84.300000
DGY9PNT01CES6H	CR_Glycine_max_FJ197993_1_1_9437_gmp1-71h23-re-7_gag(pfam037032)	94.590000	37	2	0	117	7	4	40	0.000000	82.000000
DGY9SYN02JAP6R	CR_Glycine_max_FJ197993_1_1_9437_gmp1-71h23-re-7_gag(pfam037032)	80.950000	42	8	0	3	128	3	44	0.000000	81.600000

move selected to Ids editor

Figure 8: Table with blast results. Columns of the table can be sorted by clicking on the column header.

7 GGobi window

When GGobi window is accidentally closed, it can be re-opened from the main SeqGrapheR window using *GGobi/Reopen*. When any GGobi window is still open this action will close all existing GGobi windows first and a new instance if GGobi will be created.

8 Project

The current graph with all Ids lists, the last blast results, and sequences can be saved in one file as a project to work with later using *File/Project/Save and File/Project/Open*. Note that the current graph coloring will not be preserved. Current graph image can be saved into postscript file (*File/Export/Graph image*). The current projection of the graph as seen in the ggobi graph window can be saved as a new graph in a GL file. This file is the same as the original GL graph except the coordinates of the layout are correspondingly rotated.
